# Optical Fourier Surfaces for Free-Space Computer-Generated Holography

**DOI:** 10.1021/acsnano.6c13095

**Published:** 2026-10-19

**Authors:** Yannik M. Glauser, Che-Yung Shen, David B. Seda, Çaǧatay Işil, Patrick Benito Eberhard, Hannah Niese, Juri G. Crimmann, Daniel Petter, Gabriel Nagamine, Sander J. W. Vonk, Nolan Lassaline, Aydogan Ozcan, David J. Norris

**Affiliations:** † Optical Materials Engineering Laboratory, Department of Mechanical and Process Engineering, 27219 ETH Zurich, Zurich 8092, Switzerland; ‡ Department of Electrical and Computer Engineering, 8783 University of California, Los Angeles, California 90095, United States; § Department of Bioengineering, University of California, Los Angeles, California 90095, United States; ∥ California NanoSystems Institute (CNSI), University of California, Los Angeles, California 90095, United States; ⊥ Department of Surgery, David Geffen School of Medicine, University of California, Los Angeles, California 90095, United States; # Department of Physics, Technical University of Denmark, Kongens Lyngby 2800, Denmark

**Keywords:** thermal scanning-probe lithography, computer-generated holography, Fourier engineering, iterative Fourier transform algorithm, inverse design, spectral multiplexing

## Abstract

Computer-generated holography can shape electromagnetic fields by encoding wavefront information in nanostructured surfaces. However, despite significant progress, hologram designs for visible and near-infrared wavelengths remain largely limited by fabrication constraints. Most implementations rely on lithographic methods that produce discretized surfaces that do not match the wave nature of light. Further advances would benefit from accurate, continuous (grayscale) control of interfacial profiles, which would allow straightforward design principles from Fourier optics to be applied. In this work, we introduce optical Fourier surfaces as a versatile, intuitive platform for free-space computer-generated holograms. Exploiting these arbitrarily wavy surfaces, we demonstrate three different design strategies for computer-generated holography. First, we use an analytical linear design approach based on sinusoidal lenses and gratings as fundamental holographic building blocks. Second, we enhance diffraction efficiencies through an iterative Fourier-transform algorithm. Finally, we extend our framework with differentiable inverse design, creating wavelength-multiplexed holograms that reconstruct distinct diffraction responses in a predefined image plane. Our results combine advanced thermal scanning-probe lithography with flexible design strategies to create useful structures for photonic applications, particularly in settings where rapid prototyping is crucial.

## Graphical abstract

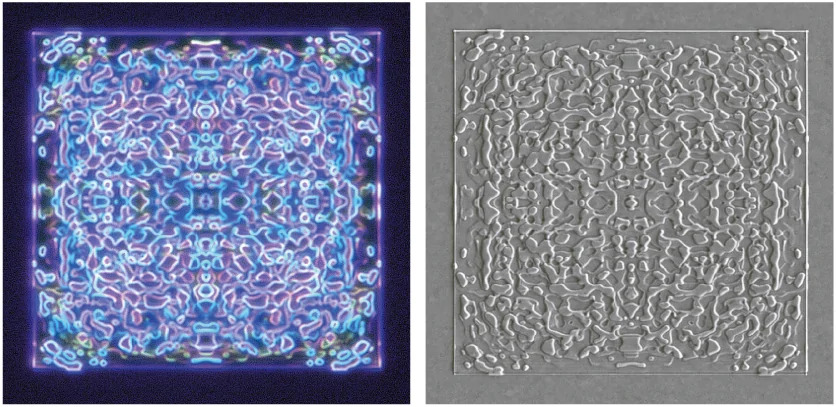

## Introduction

The ability to control light is key for many technologies, including imaging,^1,2^ augmented reality,^3^ communication,^4,5^ and computing.^6,7^ For such applications, holography presents a general framework for tailoring the optical wavefront in terms of its amplitude, phase, polarization, and spectrum. In its traditional form, holography involves a two-step process:^8,9^ (i) an interference pattern between an object and reference wave is recorded as a hologram in a medium, and (ii) this hologram is later reilluminated with the reference wave to generate an image of the original object. However, the need for a physical recording step can be limiting for applications. To avoid this, computer-generated holography (CGH) has been developed.^9^ It calculates the required hologram directly using computer algorithms, offering a route to full digital control over the information encoded in the electromagnetic field.

To implement this approach in practice, the hologram must then be fabricated from the computer-generated design. For visible and near-infrared light, the desired structures often contain surface profiles that continuously vary in depth (*z*).^10–19^ Unfortunately, most methods that tailor interfacial depth (known as grayscale lithography) have not yet demonstrated sufficient control for the required “wavy” profiles. Consequently, researchers have restricted their designs to surface profiles with only two depth levels.^20^ While, in general, this constraint reduces optical performance, properly arranged arrays of “binary” nanostructures, known as metasurfaces, can collectively provide advanced capabilities.^21,22^ These structures offer independent control over amplitude,^23–27^ phase,^21,24–28^ polarization,^23,26–29^ and/or spectrum^30^ by engineering subwavelength building blocks. However, the design process for metasurfaces is usually highly specialized and nonintuitive, relying on computationally intensive full-field simulations to achieve a design with the desired optical response. These difficulties arise because metasurfaces force binary elements to shape electromagnetic waves—a task that is presumably better matched to wavy patterns. This suggests that more effort should focus on improved grayscale solutions for CGH that combine highly accurate nanofabrication with quick and intuitive design.

Recently, thermal scanning-probe lithography (TSPL) has been introduced as a powerful method to obtain grayscale surface patterns with nanometer-scale accuracy.^31–34^ Because this technique enables surface profiles with precise depth control, it can provide arbitrarily wavy diffractive interfaces, known as optical Fourier surfaces (OFSs).^35^ To date, OFSs made by TSPL have primarily been explored in two distinct contexts. First, they have served as model systems to perform fundamental investigations of diffraction from high-fidelity sinusoidal gratings.^36^ Second, they have been incorporated into architectures that target integrated photonics, e.g., grating couplers, waveguide resonators, local sources/sensors, and optical-computing elements.^37–40^ These developments demonstrate the capabilities of high-fidelity OFSs, but their application to free-space CGH has remained largely unexplored. Owing to their direct connection to Fourier optics, OFSs provide a natural platform for realizing compact, high-fidelity holograms in the visible and near-infrared.

Here, we use TSPL to fabricate wavy diffractive surfaces in silver (Ag) for CGH. We systematically investigate and compare three complementary design strategies: (i) analytical linear design, (ii) iterative design using a generalized Gerchberg–Saxton (GS) algorithm,^41,42^ and (iii) gradient-based differentiable inverse design.^6,43,44^ While each approach offers distinct strengths and limitations, all of them can be seamlessly implemented with our OFSs. By combining these flexible design strategies with our accurate fabrication procedure, we realize compact, high-fidelity OFS holograms capable of producing sophisticated diffraction patterns.

## Results and Discussion

### Optical Fourier Surfaces as Reflective Phase Holograms

We fabricated Ag OFSs by applying a previously reported procedure.^37^ Briefly, we performed the following steps: (i) thermally sensitive poly­(phthalaldehyde) (PPA) thin films were patterned with TSPL,^31–33^ (ii) Ag was then thermally evaporated onto the PPA,^45^ (iii) the Ag replica was template-stripped off of the PPA,^46^ and (iv) the final Ag structure was cleaned with anisole (see Methods and Figure S1 in the Supporting Information). Compared to our prior work, we achieved ∼20% deeper structures (with depths of up to 350 nm) by improving the feedback calibration of the TSPL tool. The fidelity of the fabrication process was further evaluated for representative holograms obtained using each of the three design strategies. Comparison of the designed and fabricated topographies shows RMSE values of 3–23 nm (Figure S2 in the Supporting Information), demonstrating that the complex surface profiles are accurately reproduced, including for the deepest structures.

The resulting Ag OFSs act as phase-only reflective holograms by locally introducing optical-path differences to the incoming reference wavefront with free-space wavevector *k*_0_ (Figure 1a). The height profile *h*_p_(*x*, *y*) imprints a phase modulation *ϕ*(*x*, *y*) = 2*k*_0_ *h*_p_(*x*, *y*) at the sample plane. After free-space propagation of the reflected light, a complex diffraction pattern *U*(*x*, *y*) appears at a target image plane.^9,36,47,48^ In this context, CGH using OFSs involves the direct design of the phase modulation introduced by the surface profile. Even though the surface profile only alters the phase in the sample plane, manipulation of the amplitude and/or phase of the light in the far field (Fourier space) or at any arbitrary image plane is possible.^37^ However, in free space, inherent trade-offs exist between the image quality and diffraction efficiency. The spatial resolution of OFSs (down to ∼30 nm in-plane and ∼2 nm in depth for shallow structures) allows these trade-offs to be optimized.

**Figure 1:**
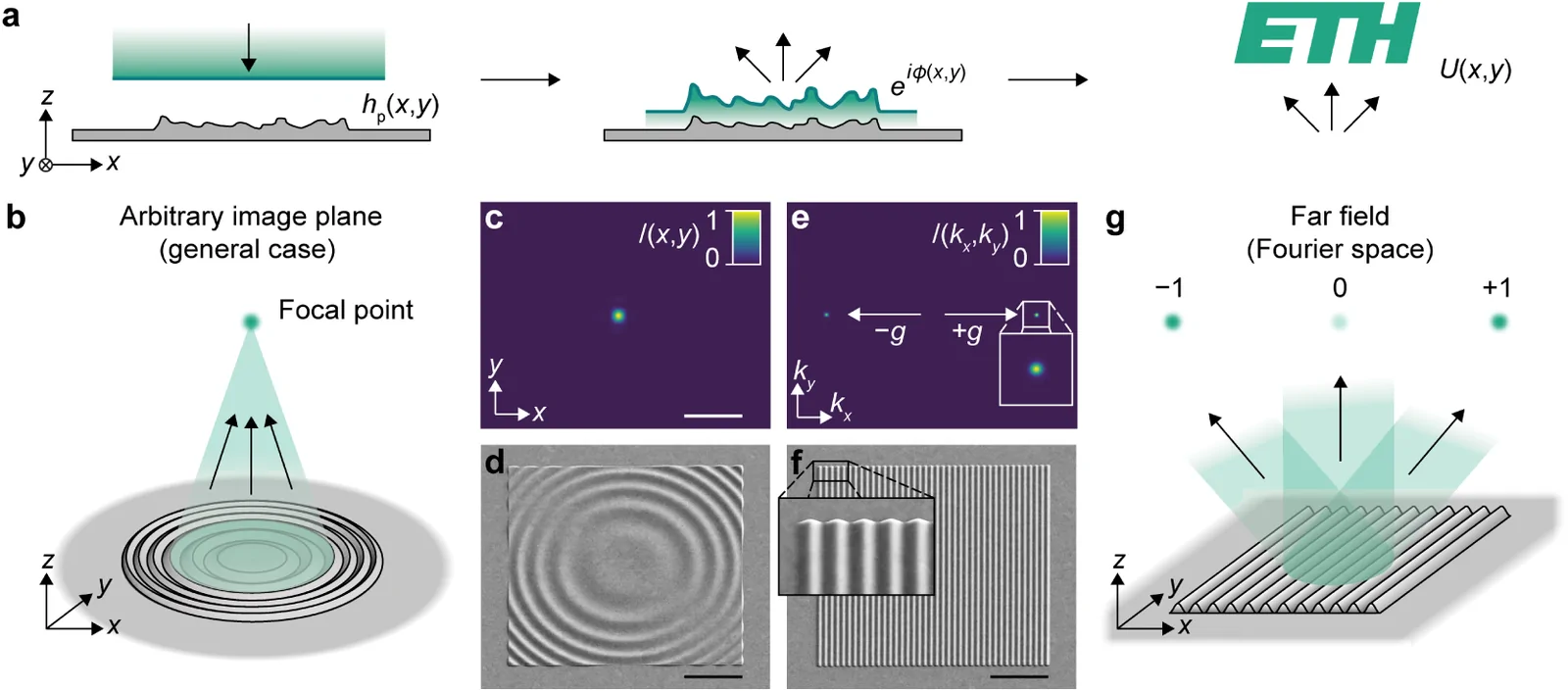
Optical Fourier surfaces (OFSs) in Ag as reflective phase**-**only holograms. (a) Computer-generated holography (CGH) with OFSs. The OFS hologram is designed and fabricated with a height profile *h*_p_(*x*, *y*) that induces a phase modulation *ϕ*(*x*, *y*) on the reflected and diffracted light in the sample plane, thereby shaping the complex wavefront *U*(*x*, *y*) in an arbitrary image plane. (b) Schematic of a sinusoidal lens with focal distance *f*, focusing light to a specific spot. (c) Experimentally measured focal spot for 532 nm light of a sinusoidal lens in Ag at a distance *f* = 100 μm. (d) Scanning electron microscopy (SEM) image of the corresponding lens. (e) Experimental far-field diffraction pattern of a sinusoidal grating with a period Λ = 1000 nm. (f) SEM image of the corresponding grating in Ag. (g) Schematic of sinusoidal OFSs diffracting light into the far field (Fourier space). SEM images in (d) and (f) were acquired at a tilt angle of 30°. The scale bar is 10 μm. Intensities in (c) and (e) are normalized to their respective maximum values and plotted on a linear scale. Logo used with permission from ETH Zurich.

Prior works have developed strategies to address CGH for surfaces that only affect the phase. Typically, these approaches exploit Fourier optics and scalar diffraction theory.^9,49^ In the most general case, the desired image is formed in an arbitrary image plane located at a specific distance from the diffractive hologram. Light propagation is analytically described using the angular-spectrum method.^9,50^ The two-dimensional wavefront at the sample plane (input plane in *x* and *y*) is decomposed into its spatial Fourier components, which then propagate in the *z* direction according to a transfer function. These propagated Fourier components are subsequently recombined to reconstruct the diffraction signal in the image plane (output plane). A special case arises when the diffraction image is formed in the far field. Light propagation to the far field (Fourier space) is then simply described by a Fourier transform of the electric field profile in the sample plane.^9^ This can be realized in an optical setup by imaging the back focal plane of a 2*f* imaging system, where *f* is the focal length. (See Methods and Figure S3 for experimental details and Sections S1.1 and S1.2 in the Supporting Information for diffraction theory.)

### Analytical Linear Design

To design OFSs for CGH, we must determine the surface profile (i.e., the phase modulation at the sample plane) that creates the desired diffracted signal at the output plane. For shallow profiles, we can follow a linear approach.^35,37^ Namely, we can simply superpose individual diffractive elements to create the desired output. Mathematically, this follows from a first-order Taylor expansion of the phase *e^iϕ^* ≈ 1 + *iϕ*. This approximation is valid for small phase modulations and inherently neglects higher-order contributions.

For our fundamental diffractive element, we consider sinusoidal lenses (Fresnel zone plates, Figure 1b) or sinusoidal gratings (Figure 1g). Each produces distinct, point-like diffraction features: a tight focus and discrete diffraction spots, respectively (Figure 1b–g). Superposition of these building blocks enables the construction of more advanced diffracted images. In contrast, the superposition of other elements, such as blazed Fresnel lenses or blazed gratings, simply results in another lens or grating with only one diffracted feature, offering no additional functionality. The direct superposability of the sinusoidal elements therefore makes them particularly useful as building blocks for constructing more complex optical functions (Section S1.3 and Figure S4 in the Supporting Information).

TSPL allows us to exploit such wavy sinusoidal lenses (Figure 1d) and sinusoidal gratings (Figure 1f) as building blocks. Even its high lateral spatial resolution, down to ∼30 nm for shallow structures, is beneficial. While in principle high spatial frequencies (corresponding to periods, Λ, shorter than the optical wavelength, λ) only generate evanescent waves that do not propagate to the far field, they still influence the diffraction output through higher-order convolution effects. We show analytically (Section S1.4) that all spatial frequencies contribute to the output signal. However, the contributions of structural features with periods Λ < λ/4 are small (Figure S5 in the Supporting Information).

While the linear approach is valid for any image plane, we restrict our discussion now to the far field. In this case, the inverse Fourier transform $F^{-1}$ of a desired complex-valued diffraction image yields the corresponding surface profile (up to a scaling factor), enabling the direct and intuitive design of OFS holograms (Figure 2a). To ensure that $F^{-1}$ results in a real-valued phase map that can be introduced by a single reflective surface profile, the tunable part of the desired diffraction pattern F{ϕ}=U(x,y) needs to be Hermitian, with *U*(−*x*, −*y*) = *U* ^*^(*x*, *y*). This requirement arises because only conjugate-symmetric Fourier spectra correspond to real-valued functions in real space (see Section S1.5 in the Supporting Information).

**Figure 2:**
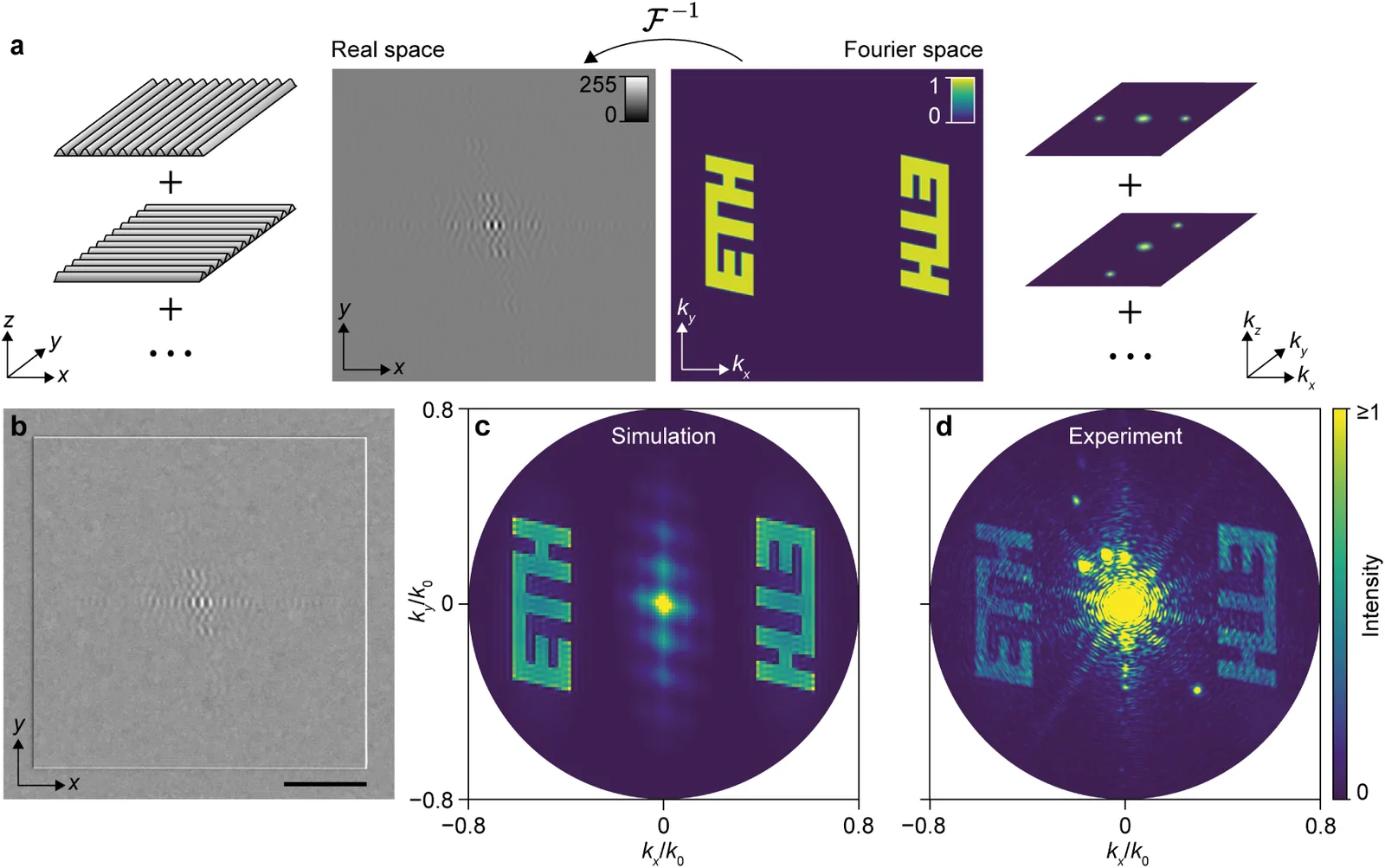
Linear design approach for far**-**field holography with OFSs. (a) Inverse Fourier transform of the target diffraction pattern (complex-valued) in Fourier space to obtain the corresponding OFS design in real space (sample plane). We use a reference wave at normal incidence with *λ* = 532 nm. The OFS is rescaled to a grayscale map for TSPL (8-bit precision). (b) SEM image of the fabricated OFS hologram in Ag, designed to produce a diffraction image in the form of the logo of ETH Zurich. During fabrication, the design in (a) was converted to a surface profile with a target depth of *λ*/2 = 266 nm. The scale bar is 10 μm. (c) Simulated and (d) experimentally measured far-field diffraction intensities in Fourier space (i.e., the back focal plane of the objective with a numerical aperture, NA of 0.8). Intensities in (c) and (d) are normalized to the respective maximum values of the diffraction image (excluding the 0th-order specular reflection) and plotted on a linear scale. The simulation is based on scalar diffraction theory. Logo used with permission from ETH Zurich.

We first investigate OFSs formed by azimuthal superpositions of sinusoidal profiles (see Section S1.6 and Figures S6 and S7 in the Supporting Information). Each sinusoid corresponds to a specific spatial frequency that produces a pair of diffraction spots in the far field (−1st and +1st orders symmetrically arranged around the 0th-order spot). Therefore, superposing many sinusoids enables us to diffract light simultaneously into various directions, forming a desired pattern in the far field (Fourier space). In particular, holograms with useful diffraction patterns for photonic applications^51^ can be generated.

For example, we fabricated an OFS hologram that projects the ETH logo (Figure 2b). To prevent overlap with the specular reflection, the target image is offset in Fourier space. The specular reflection was eliminated using a beam block. The corresponding simulated and experimentally measured far-field intensity patterns are shown in Figure 2c,d. The results are in good agreement with the targeted diffraction image. Analogous results for a grayscale image (a cube with facets of varying intensities) are presented in Figure S8 in the Supporting Information. Note that as more sinusoids are superposed, the contribution from each becomes weaker (Figure S7 in the Supporting Information). Thus, the overall intensity in Figure 2d remains relatively low compared to the specular reflection. The simulated and experimental efficiencies were 0.18 and 0.11% per image (0.36 and 0.21% for both combined), respectively (Figure 2c,d and Figure S9 in the Supporting Information). To illustrate the benefit of the continuous depth control provided by OFSs, we additionally compare this wavy hologram with binarized versions of the same design (Figure S10).

Fortunately, in some applications, the overall efficiency is not the most important characteristic. Instead, the image quality in terms of its uniformity and sharpness plays a more critical role.^52,53^ For our structures, the obtained images are uniform in intensity. This uniformity is consistent with the flat phase profile expected from the Fourier-based design, as supported by the simulated phase distributions in Figure S11 in the Supporting Information. Such phase control reduces interference-induced speckle that can cause nonuniform diffraction images, a common problem in CGH.^54^

To enhance the diffraction efficiency of the hologram within the linear approach, we can rescale the surface profile to boost the amplitude of each sinusoidal component. By keeping the total pattern depth fixed, this scaling process leads to cropping of the dominant sinusoidal features in the center of the OFS (Figure S12 in the Supporting Information). As the scaling factor increases, the diffraction efficiency of the hologram improves. However, with increasing pattern depth, the phase modulation progressively departs from the small-phase regime assumed by the linear approach. Effects due to higher-order diffraction become more dominant, resulting in images with reduced uniformity and more pronounced contours.

Although we have so far focused on far-field images, the linear design approach can also be applied to arbitrary image planes. However, in these cases, the fundamental building blocks are sinusoidal lenses, each focusing light to a specific location in the image plane (Figure 1b–d). By superposing these lenses, desired focal patterns can be created.^37^

### Iterative Design

While the linear approach is intuitive and offers control over amplitude and phase, some applications require higher diffraction efficiencies. Deeper profiles can provide this efficiency, but the relationship between the profile and signal is then nonlinear. Namely, the superposition of two patterns with phases *ϕ*_1_(*x*, *y*) and *ϕ*_2_(*x*, *y*) in the surface results in a product of complex exponentials $e^{i\left(\phi_{1}+\phi_{2}\right)}=e^{i\phi_{1}}e^{i\phi_{2}}$ . In the far field, this causes a convolution of the individual diffraction signals,

$$F\left\{e^{i\phi_{1}+i\phi_{2}}\right\}=F\left\{e^{i\phi_{1}}\right\}\;\times \;F\left\{e^{i\phi_{2}}\right\}\neq F\left\{e^{i\phi_{1}}\right\}+F\left\{e^{i\phi_{2}}\right\}$$ 1

In other words, the linear superposition of surface profiles does not yield a linear superposition of the corresponding diffraction patterns, but rather their convolution.

For this case, iterative design strategies can be applied.^49^ This results in less intuitive designs that impose fewer constraints, e.g., no Hermitian symmetry is required. The most commonly used methods for far-field holography are iterative Fourier-transform algorithms (IFTAs). They are also more broadly exploited in electron microscopy, astronomy, and crystallography.^41,42^ Essentially, they allow phase information to be reconstructed from intensity measurements. Consequently, they are often referred to as phase-retrieval algorithms. Variations of IFTAs include steepest-descent, input–output, and error-reduction methods, with the Gerchberg–Saxton (GS) algorithm the most widely used among the latter.^41,42,55^

To complement our linear design approach, we investigated a generalized form of the GS algorithm that iteratively refines the complex wavefront at the object domain (sample plane, real space) and the Fourier domain (image plane, Fourier space). In between, both object- and Fourier-domain constraints are imposed. See Section S1.7 and Figure S13 in the Supporting Information for a detailed description of the procedure.

We employed this iterative approach to project the ETH logo and a grayscale cube. Without intervention, the GS algorithm introduces discontinuities in the surface profile (phase jumps from 0 to 2*π*), which are not possible to fabricate with TSPL. To remove these, we postprocessed the design by applying a Gaussian filter (standard deviation *σ* = 60 nm) to smooth the surface and generate the final design for fabrication. Figure 3a,b shows the fabricated holograms, which exhibit different structural features compared to the linear designs. Figure 3c,d plots the corresponding far-field diffraction patterns, alongside a simulation based on scalar diffraction. For the ETH logo, the target images are well reconstructed in both simulation and experiment with efficiencies of 57.6 and 32.9%, respectively. The measured value is significantly higher than that from the linear approach. However, the diffraction images exhibit speckle patterns and reduced uniformity. As discussed above, these artifacts are a consequence of the unconstrained phase in the Fourier domain, which can introduce undesired interference.^54^

**Figure 3:**
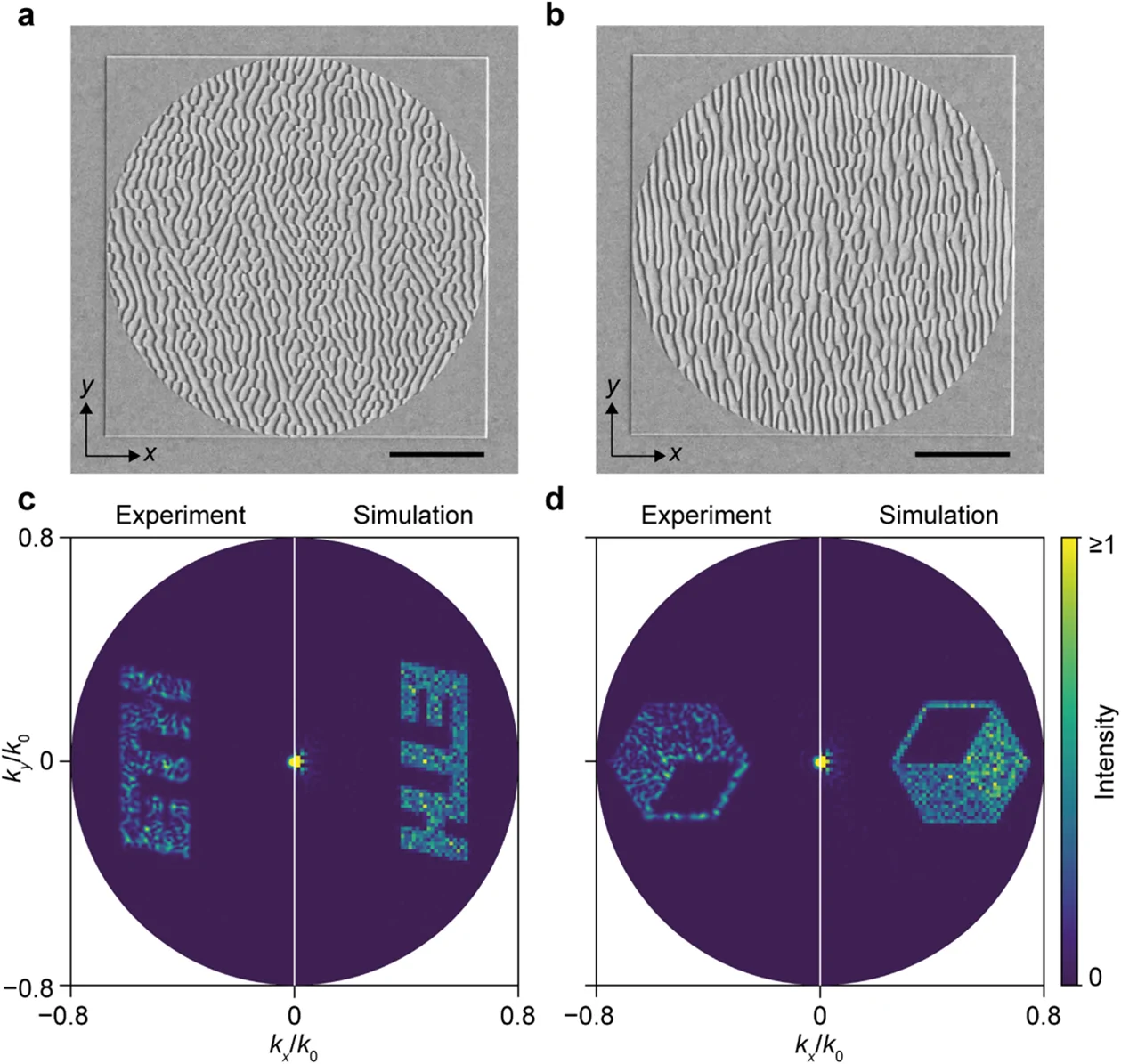
Iterative design approach based on the Gerchberg–Saxton (GS) algorithm. (a) and (b) SEM images of fabricated OFS holograms in Ag, designed with an iterative Fourier transform algorithm (IFTA) to generate the ETH logo and a grayscale cube, respectively. Both structures have a lateral size of 40 μm × 40 μm (scale bar: 10 μm) and target depths of 228 nm (ETH logo) and 236 nm (cube), tailored to incident light with *λ* = 532 nm. (c) and (d) Simulated and experimentally measured far-field diffraction intensity patterns (NA of 0.8). Intensities for simulation and experiment are normalized to the respective maximum values of the diffraction image (excluding the 0th-order specular reflection) and plotted on a linear scale. Data were acquired under the same conditions as in Figure 2 (see Supporting Information for details). Logo used with permission from ETH Zurich.

To improve uniformity, several variations of the GS algorithm have been reported that constrain the phase of the wavefront in the Fourier domain.^54,56^ We tested this strategy by adapting the iterative algorithm for our OFS platform. It produced designs similar to those obtained directly from the linear approach (see Figure S14 in the Supporting Information). In these cases, the final iteration largely dictated the design outcome, as it applied the inverse Fourier transform after enforcing both amplitude and phase constraints in the Fourier domain (analogous to the linear approach). This observation highlights that the surface designs produced by different approaches converge, when similar constraints are applied.

### Differentiable Inverse Design

To further explore the capabilities of our OFS holograms, we pursued a stochastic gradient descent-based differentiable inverse-design framework that used deep learning for diffractive feature optimization.^6,43^ This enabled access to a broader set of design solutions that overcome the limitations of linear or standard iterative design approaches. We leveraged it to realize wavelength-multiplexed holograms that project diffraction images at arbitrary image planes, moving beyond the conventional far-field regime. More specifically, the design algorithm optimized the surface profile at the sample plane such that, upon free-space propagation, it generated the desired intensity patterns, at specific wavelengths, in a target image plane located at a defined distance from the OFS hologram. During the gradient-based optimization of diffractive features, a mean-squared-error (MSE)-based loss function between the output intensity profiles and their corresponding ground truth images was minimized using error backpropagation through the differentiable propagation model; see the Supporting Information for details.^57^ In addition to the image-fidelity term, the loss function incorporated output diffraction efficiency-based constraints and fabrication-aware penalty terms that enforced a maximum allowable profile slope of 1 (45°), thereby reducing potential discrepancies between simulations and experimental results (see Section S1.8 in the Supporting Information). To further improve robustness, a Gaussian filter (*σ* = 320 nm) was also applied to the surface profile during optimization. We assumed plane wave illumination, which is experimentally approximated by a Gaussian beam with a large beam diameter (see Methods and Figure S3b in the Supporting Information).

As a proof of concept, we designed, fabricated, and optically characterized a wavelength-multiplexed hologram that reconstructs distinct images at three different wavelengths (*N_w_* = 3). Figure 4a,b shows the original structure in PPA and the OFS hologram in Ag, respectively. The corresponding target images in Figure 4c,f,i are plotted alongside the simulated images in Figure 4d,g,j, which are calculated based on the angular-spectrum method.^9,50^ They represent handwritten digits 0, 1, and 2 from the Modified National Institute of Standards and Technology (MNIST) data set (each image with 25 × 25 pixels) assigned to wavelengths *λ* = 450, 575, and 700 nm, respectively, reconstructed at an image plane located 200 μm away from the hologram. Figure 4e,h,k shows the experimentally measured diffraction images for the different wavelengths. The desired images are diffracted into a field of view (FOV) of 50 μm × 50 μm (indicated by the dashed square in Figure 4e) with residual intensity distributed around this region. While good agreement between the target and diffracted images is observed, the experimental results exhibit lower overall intensity and increased crosstalk between the wavelength channels compared to the simulations. Quantitative values for diffraction efficiency and image quality (peak signal-to-noise ratio, PSNR) are provided in Table S1 in the Supporting Information. These discrepancies are likely due to fabrication imperfections, experimental setup-related artifacts and potential misalignments. In addition, we observe speckles within the reconstructed images, which we attribute to the unconstrained phase of the wavefront in the design process. To further quantify the spectral response of the wavelength-multiplexed hologram, we analyzed the crosstalk between the three target images and their wavelength dependence in both experiment and simulation (Figure S15).

**Figure 4:**
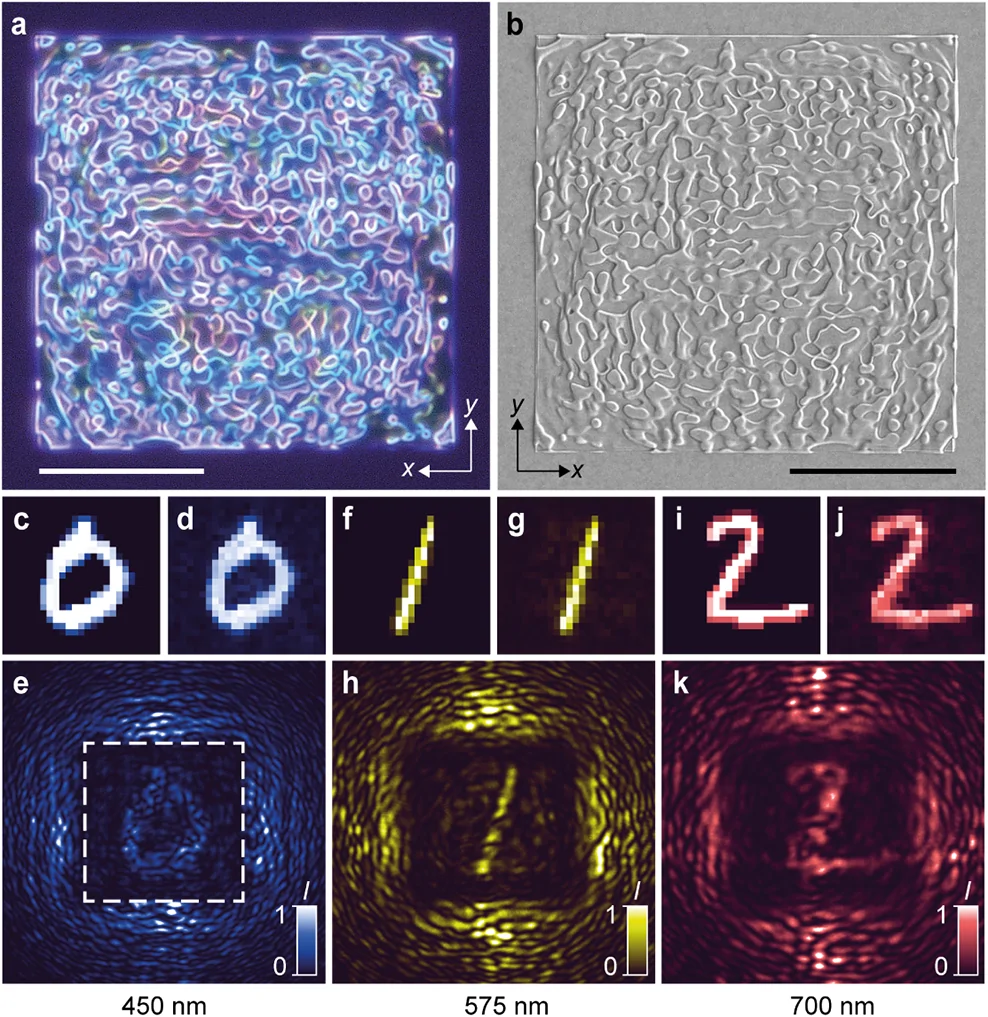
Wavelength-multiplexed OFS hologram (*N_w_* = 3). (a) Dark-field (DF) optical image of a 3-fold wavelength-multiplexed hologram in PPA, patterned with a pixel size of 40 nm × 40 nm. (b) SEM image of the corresponding structure in Ag with a lateral size of 50 μm × 50 μm and a depth of 350 nm. The structure is designed to diffract light at three different wavelengths into distinct diffraction images at 200 μm distance from the sample plane. Scale bars in (a) and (b) are 20 μm. (c), (f), and (i) Target diffraction intensity patterns at the image plane for *λ* = 450, 575, and 700 nm, respectively. The images correspond to handwritten digits 0, 1, and 2 from the MNIST data set. (d), (g), and (j) Simulated diffraction intensities for the respective wavelengths based on simulations using the angular-spectrum method. (e), (h), and (k) Experimentally measured diffraction intensities of the diffraction images for the three different wavelengths. The dashed square in (e) indicates the field of view (FOV) of 50 μm × 50 μm for which the OFS hologram was designed. Intensities in (c)–(k) are normalized to the respective maximum values within the FOV and plotted on a linear scale. (c)–(e), (f)–(h), and (i)–(k) share the same intensity scale bars, respectively.

For deeper insight into the behavior of a wavelength-multiplexed hologram, we systematically analyzed several key design parameters: (i) the distance between the OFS hologram and image planes, (ii) the spectral spacing, and (iii) the number of multiplexed channels *N_w_* (see Figures S16 and S17 in the Supporting Information). These studies revealed clear trade-offs in the information content encoded in a single OFS, influenced by, e.g., image complexity, reconstruction quality, crosstalk, diffraction efficiency, robustness, and fabrication feasibility. In particular, increasing the number of wavelength channels places greater demands on the surface profile, making it necessary to reduce the complexity of each target image to maintain reliable reconstruction across all channels. Guided by this analysis, we found that four-wavelength (*N_w_* = 4) spectral multiplexing could be improved by reducing the target diffraction-image resolution from 25 × 25 to 7 × 7 pixels, thereby relaxing the information-density requirements imposed on a single OFS hologram. To experimentally demonstrate this design strategy, we fabricated some representative four-channel OFS holograms. Figure 5a–c shows the patterned structures in PPA for three different image series: geometric shapes (squares and rectangles), the letters “ETHZ,” and the letters “UCLA,” respectively. The corresponding OFS holograms in Ag are shown in Figure 5d–f. Simulated and experimentally measured diffraction images at an image plane located 200 μm from the sample plane are plotted in Figure 5g–l for wavelengths *λ* = 440, 540, 640, and 740 nm. The measured intensity patterns inside the FOV were binned to match the simulation resolution (see raw experimental data in Figure S18 in the Supporting Information). The corresponding efficiencies and PSNR values are summarized in Table S2 in the Supporting Information. Collectively, these results demonstrate that gradient-based inverse design can flexibly balance image complexity, number of spectral channels, diffraction efficiency, and fabrication constraints, providing a general strategy for encoding arbitrary wavelength-multiplexed optical functions into an OFS hologram.

**Figure 5:**
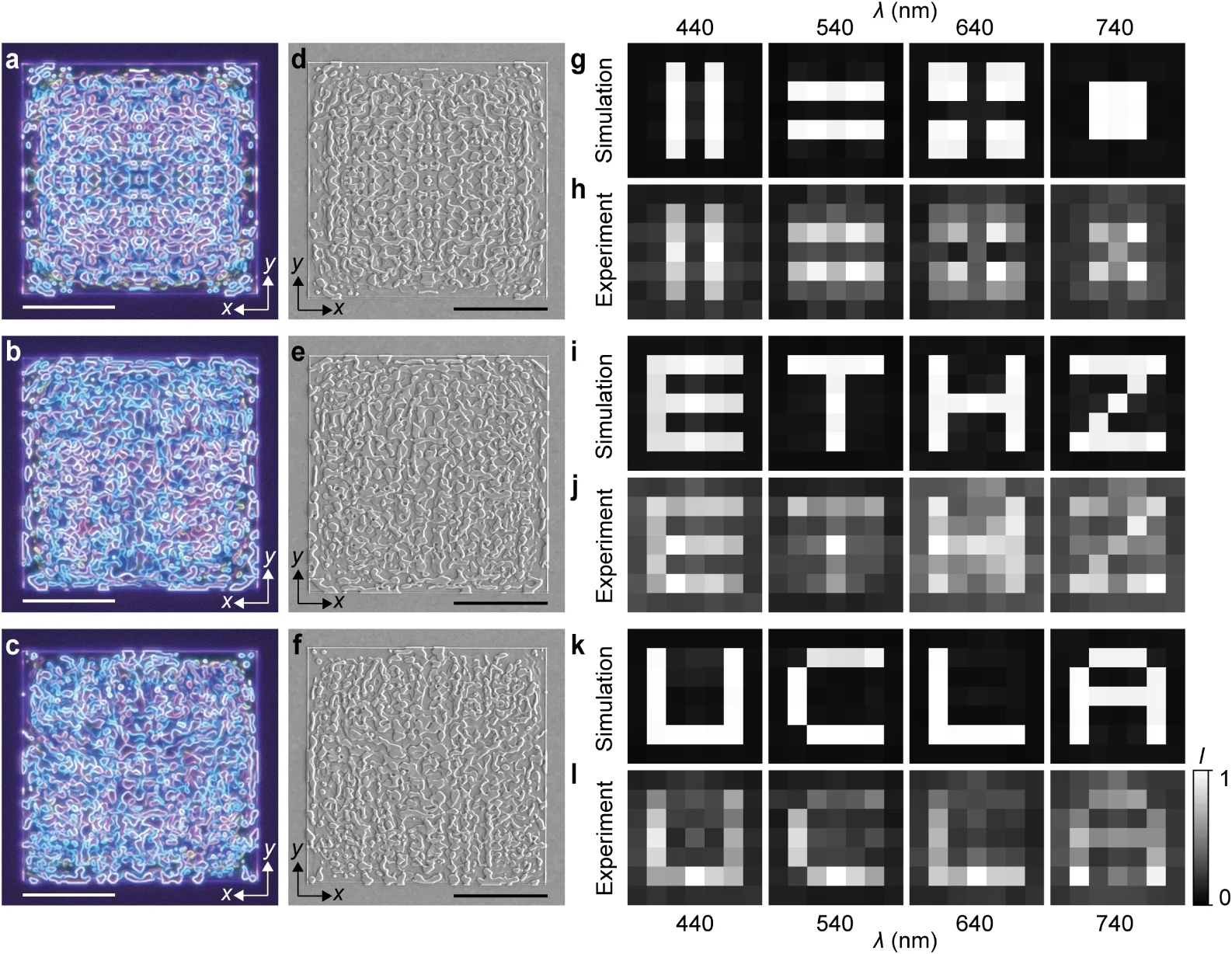
Wavelength**-**multiplexed OFS holograms (*N_w_* = 4) with reduced image resolution. (a)–(c) DF images of the 4-fold wavelength-multiplexed holograms in PPA for three different image series: geometric shapes (squares and rectangles), the letters “ETHZ,” and the letters “UCLA,” respectively. (d)–(f) SEM images of the corresponding OFS holograms fabricated in Ag. These structures are designed to diffract light at wavelengths *λ* = 440, 540, 640, and 740 nm into distinct diffraction images at an image plane located 200 μm above the sample plane. They have a lateral size of 50.4 μm × 50.4 μm and a depth of 350 nm. Scale bars in (a)–(f) are 20 μm. (g)–(l) Simulated and experimentally measured diffraction intensities within the FOV, which spans 50.4 μm × 50.4 μm. The experimental data were binned to 7 × 7 pixels for direct comparison with the simulations. Each data set in (g)–(l) was normalized to its respective maximum value and plotted on the same linear scale. The raw experimental data are provided in Figure S18 in the Supporting Information.

## Conclusions

In this work, we introduce high-fidelity OFSs as a versatile platform for free-space CGH in the visible and near-infrared regime. Combining nanometer-scale fabrication through TSPL with analytical and data-driven design approaches, we demonstrated compact holograms that encode diffraction patterns at high spatial resolution. Our improved fabrication process—based on an optimized feedback calibration of the TSPL tool—enables high-fidelity OFSs with increased depth. This advancement supports the production of holograms across a broader design space. We investigated design methodologies with varying levels of complexity and reconstruction performance. Starting with sinusoidal gratings and lenses as intuitive building blocks, we developed a linear superposition framework that provides direct analytical control over amplitude and phase in the far field. To boost diffraction efficiencies, we applied a generalized GS algorithm, which relaxes first-order approximations at the cost of phase control, leading to reduced homogeneity in the reconstructed images. Finally, we employed gradient-based differentiable inverse design to create wavelength-multiplexed holograms that project distinct patterns at different wavelengths onto a predefined image plane. We experimentally demonstrated up to 4-fold multiplexing. This approach effectively adapts to variations in image resolution, spectral spacing, number of wavelength channels, or distance between sample and image plane. The key characteristics, advantages, and limitations of the three design strategies are summarized in Table S4 in the Supporting Information. Altogether, the design flexibility and simplicity of OFSs combined with their accurate fabrication procedure provide benefits for applications in rapidly evolving fields where fast prototyping is essential.

## Methods

### Design of Fourier Surfaces and Bitmap Generation

Our designs of the OFS holograms were generated either from analytical functions [for gratings, lenses, and (quasi)­crystals] or from one of the three design strategies outlined above: linear design, iterative design via IFTAs, or gradient-based differentiable inverse design. The feasibility of these designs is governed by the limitations of TSPL fabrication, including constraints on maximum lateral dimensions, depth, and gradient. While the lateral OFS dimension is limited by the piezo stage scan range of ∼50 μm, our experiments indicate that surface slopes exceeding ∼1 (45°) are currently not reproducible. All our design strategies explicitly incorporate these fabrication constraints.

Each hologram design is represented by an 8-bit map. We use pixel sizes down to 20 nm × 20 nm. Before loading the final designs into the TSPL tool, they are inverted along the *z* axis and mirrored in the *yz* plane to account for template stripping during fabrication.

### Fabrication Procedure

The detailed fabrication procedure has been reported previously.^37^ For TSPL, PPA was used as a thermal resist to reduce tip contamination and increase sample yield compared with other previously used resist materials.^35^ In contrast to earlier work,^35,36^ we additionally performed a cleaning step in anisole (AR 600-02, Allresist) for 2 min to remove residual PPA on the Ag. This enabled the fabrication of significantly larger and deeper structures without PPA contamination.

We also improved the feedback calibration of the TSPL tool to increase the accessible pattern size and depth. To this end, the feedback was trained by patterning structures with gradually increasing depths until the desired depth, up to 350 nm, was reached. Depending on the complexity of the pattern, the writing time per pixel was increased from the default value of 25 to 50 μs, while the in situ topography readout was upsampled by a factor of 2.

Hologram designs were analytically generated within seconds using the linear approach, or iteratively within minutes using the GS algorithm, and within several hours using gradient-based inverse design. Individual structures were patterned by TSPL within approximately 30 min, depending on the sampling and feedback settings. Chips containing tens of structures were subsequently processed within 1 day, including Ag evaporation and template stripping.

### Characterization of Surface Topography

The accuracy of our fabrication procedure has been demonstrated in previous studies.^35,36,58^ To verify the updated process, which includes additional anisole cleaning, we used a reference structure: a single-sinusoidal profile with period Λ = 600 nm, amplitude *A* = 25 nm, and lateral dimensions of 9 μm × 9 μm. The sinusoidal structure is surrounded by a 1-μm-wide patterned frame (see Figure S19 in the Supporting Information).

First, we investigated the pattern transfer from PPA to Ag by comparing the original structure in PPA with the final structure in Ag. Both topographies were measured by atomic force microscopy (AFM). We leveled the measured profiles by performing a two-dimensional planar fit based on the unpatterned areas surrounding the OFS. Afterward, we fit a sinusoidal function to the patterned surface profile. The characterization was performed using a function-fitting software package (FunFit).^59^ The corresponding data (fit parameters) can be found in Table S3 in the Supporting Information. The results confirm that the surface profile was not changed during pattern transfer to Ag (deviations in periods and amplitudes are ∼1 nm). Moreover, we compared the roughness for the patterned and unpatterned areas in Ag. In line with our previous findings,^35^ we observed that the patterned areas exhibited smaller surface roughness (1.4 versus 1.9 nm root-mean-square roughness).

Second, we compared the topography data obtained from the TSPL tool with the AFM topography data of the same structure in PPA. We found good agreement between both methods (see fit parameters in Table S3 in the Supporting Information).

We concluded that (i) the pattern transfer from PPA to Ag does not alter the surface structure, and (ii) the TSPL tool provides topography data comparable to AFM topography measurements. Thus, the TSPL data obtained during patterning of the PPA was sufficient for the surface characterization of the OFS holograms in Ag. This is consistent with our previous work.^36^

### Optical Measurements

For both the far-field diffraction experiments and those with an image plane at an intermediate distance from the sample plane, we used the same optical setup in two slightly different configurations (Figure S3 in the Supporting Information). The home-built setups are based on an inverted microscope body (Eclipse Ti–U, Nikon).

As a coherent light source, we used a supercontinuum laser (SuperK Fianium, NKT Photonics) combined with a tunable band-pass filter (wavelength range 400–1000 nm, line width ∼1.5 nm; LLTF Contrast, NKT Photonics). While the wavelength for the far-field experiments was fixed to 532 nm, the wavelength was adjusted for the spectral-multiplexing experiments. The laser beam was collimated with a 10× objective [TU Plan Fluor, numerical aperture (NA) of 0.3; labeled L1 in Figure S3], after being launched from an optical fiber (A502-010-110, NKT Photonics). The light then passed through a 750 nm short-pass filter (FESH0750, Thorlabs; SP) to reject residual near-infrared light coupled out by the tunable band-pass filter. Next, the light was passed through a 90:10 beamsplitter (BSN10R, Thorlabs; BS1), a linear polarizer (WP25M-VIS, Thorlabs; P1), several mirrors, a defocusing lens (AC254-400-A-ML, Thorlabs; L2), and a 50:50 beamsplitter (AHF analysentechnik; BS2) before being focused onto the back focal plane of a 50× objective (TU Plan Fluor, NA of 0.8; L3) to achieve wide-field illumination. This resulted in a broad Gaussian beam at normal incidence (best experimental approximation of a plane wave), which was reflected from the OFS hologram. A power meter (PM100D, Thorlabs; PM) was used to monitor laser-power fluctuations over time.

To measure the far-field response of the holograms, we used a Fourier microscope configuration.^60^ While the OFS hologram was positioned in the front focal plane of the objective (real plane), emanating angles were focused to points on the back focal plane (Fourier plane, representative of the far field). The back focal plane was relayed to a digital camera (Zyla PLUS sCMOS, Andor) after passing lenses L4–L7 (tube lens, Nikon; AC254-200-A-ML, Thorlabs; AC254-200-A-ML, Thorlabs; AC508-200-A-ML, Thorlabs, respectively), an iris, and another linear polarizer (WP25M-VIS, Thorlabs; P2). To collect only light that interacted with the OFS, we placed the iris in a real plane and adjusted its diameter to match the structure side length.

To measure the diffraction response in an image plane at a specific distance from the sample plane, we defocused the sample by the desired distance and removed the Fourier lens L6. In this configuration, the desired image plane was in focus, representing a real plane in the imaging system which is mapped onto the camera. The iris was removed from the collection path of the setup.

The polarization of the incident and collected light could be independently analyzed with the polarizers (P1, P2). Because plasmonic coupling effects were negligible for our OFS holograms (p-polarized light can couple to surface plasmon polaritons under momentum-matching conditions^35,36,61^ ), we did not observe significant differences in diffraction response for different input polarizations. Therefore, both polarizers were fixed to pass light polarized along the *x* axis.

Precise alignment of the optical setup is essential for reliable diffraction measurements. We followed the same alignment procedure described previously.^36^ This procedure helped to ensure that the measured signals originated from the OFS holograms rather than from misalignment, thereby improving reproducibility and reducing systematic errors.

### Characterization of Diffraction Images

The diffraction efficiencies of the simulated and experimentally measured far-field holograms (data in Figures 2c,d and 3c,d) were determined by integrating the intensity counts over all the pixels contributing to the diffraction image. These integrated counts were then divided by the total measured counts to obtain the efficiency (see Figure S9 in the Supporting Information).

The diffraction efficiencies and PSNR values for the wavelength multiplexed holograms (data in Figures 4c–k and 5g–l) were calculated based on eqs S59 (for *N_w_* = 1) and S61 in the Supporting Information. Because the input intensity distribution on the OFS hologram is difficult to access experimentally, the diffraction efficiency for each wavelength was determined by normalizing the intensity counts within the FOV to the total detected counts within the collection NA. Consequently, this quantity represents the fraction of the collected optical power directed into the desired FOV, rather than an absolute diffraction efficiency relative to the incident power. Light diffracted or scattered outside the collection NA is not accounted for. In this evaluation, absorption losses in Ag (estimated to be a few percent across the considered wavelength range^35^) were not included.

The efficiency and PSNR values are summarized in Tables S1 and S2 in the Supporting Information. The experimentally measured diffraction efficiencies are higher than the simulated values, particularly for wavelengths near the center of the spectral range. This difference likely arises from increased crosstalk between channels in the experiments, which introduces additional signal within the FOV. While this boosts the measured efficiency, it reduces the PSNR.

## Supplementary Material



## Notes

Discussion of scalar diffraction theory and paraxial diffraction models (Sections S1.1 and S1.2). Additional information on fundamental diffractive building blocks (Section S1.3) and subwavelength features (Section S1.4). Further explanations on the different design approaches (Sections S1.5–S1.8). Additional figures and tables (Sections S2 and S3) discussing the fabrication process, the optical setup, and additional simulated and experimental data (PDF)

## References

[1] Rogers E. T. F., Lindberg J., Roy T., Savo S., Chad J. E., Dennis M. R., Zheludev N. I. (2012). A Super-Oscillatory Lens Optical
Microscope for Subwavelength Imaging. Nat. Mater..

[2] Zheludev N. I., Yuan G. (2022). Optical Superoscillation
Technologies Beyond the Diffraction Limit. Nat.
Rev. Phys..

[3] Xiong J., Hsiang E.-L., He Z., Zhan T., Wu S.-T. (2021). Augmented
Reality and Virtual Reality Displays: Emerging Technologies and Future
Perspectives. Light: Sci. Appl..

[4] Marchetti R., Lacava C., Carroll L., Gradkowski K., Minzioni P. (2019). Coupling Strategies for Silicon Photonics Integrated
Chips. Photonics Res..

[5] Hu Y. Y., He W. C., Sun Y. H., Yi Q. Y., Xing S. Z., Yan Z. W., Xia L. P., Li T., Zhou P. J., Zhang J. W., Shen L., Zou Y. (2023). High-Efficient
Subwavelength
Structure Engineered Grating Couplers for 2-*μ*m Waveband High-Speed Data Transmission. Opt.
Express.

[6] Lin X., Rivenson Y., Yardimci N. T., Veli M., Luo Y., Jarrahi M., Ozcan A. (2018). All-Optical
Machine Learning Using
Diffractive Deep Neural Networks. Science.

[7] Hu J. T., Mengu D., Tzarouchis D. C., Edwards B., Engheta N., Ozcan A. (2024). Diffractive Optical
Computing in Free Space. Nat. Commun..

[8] Saleh, B. E. A. ; Teich, M. C. Fundamentals of Photonics; John Wiley & Sons Inc., 2019.

[9] Goodman, J. W. Introduction to Fourier Optics; W. H. Freeman and Company, 2017.

[10] Rochon P., Batalla E., Natansohn A. (1995). Optically
Induced Surface Gratings
on Azoaromatic Polymer Films. Appl. Phys. Lett..

[11] Kim D., Tripathy S., Li L., Kumar J. (1995). Laser-Induced Holographic
Surface Relief Gratings on Nonlinear Optical Polymer Films. Appl. Phys. Lett..

[12] Lee S., Kang H. S., Park J. K. (2012). Directional Photofluidization Lithography:
Micro/Nanostructural Evolution by Photofluidic Motions of Azobenzene
Materials. Adv. Mater..

[13] Priimagi A., Shevchenko A. (2014). Azopolymer-Based
Micro- and Nanopatterning for Photonic
Applications. J. Polym. Sci., Part B: Polym.
Phys..

[14] Reda F., Salvatore M., Astarita M., Borbone F., Oscurato S. L. (2023). Reprogrammable
Holograms from Maskless Surface Photomorphing. Adv. Opt. Mater..

[15] Lim Y., Kang B., Hong S. J., Son H., Im E., Bang J., Lee S. (2021). A Field Guide to Azopolymeric
Optical
Fourier Surfaces and Augmented Reality. Adv.
Funct. Mater..

[16] Berdin A., Rekola H. T., Priimagi A. (2024). Complex Fourier Surfaces
by Superposition
of Multiple Gratings on Azobenzene Thin Films. Adv. Opt. Mater..

[17] Gao J., Chen P., Wu L., Yu B., Qian L. (2021). A Review on
Fabrication of Blazed Gratings. J. Phys. D:
Appl. Phys..

[18] Cheong Y. Z., Thekkekara L., Bhaskaran M., Del Rosal B., Sriram S. (2024). Broadband Diffractive
Neural Networks Enabling Classification
of Visible Wavelengths. Adv. Photonics Res..

[19] Kim J., Joy D. C., Lee S. Y. (2007). Controlling Resist Thickness and
Etch Depth for Fabrication of 3D Structures in Electron-Beam Grayscale
Lithography. Microelectron. Eng..

[20] Lee W.-H. (1979). Binary
Computer-Generated Holograms. Appl. Opt..

[21] Yu N., Genevet P., Kats M. A., Aieta F., Tetienne J.-P., Capasso F., Gaburro Z. (2011). Light Propagation
with Phase Discontinuities:
Generalized Laws of Reflection and Refraction. Science.

[22] Yu N., Capasso F. (2014). Flat Optics
with Designer Metasurfaces. Nat. Mater..

[23] Song Q., Baroni A., Wu P. C., Chenot S., Brandli V., Vézian S., Damilano B., de Mierry P., Khadir S., Ferrand P., Genevet P. (2021). Broadband Decoupling
of Intensity and Polarization with Vectorial Fourier Metasurfaces. Nat. Commun..

[24] Overvig A. C., Shrestha S., Malek S. C., Lu M., Stein A., Zheng C., Yu N. (2019). Dielectric Metasurfaces
for Complete
and Independent Control of the Optical Amplitude and Phase. Light: Sci. Appl..

[25] Liu M., Zhu W., Huo P., Feng L., Song M., Zhang C., Chen L., Lezec H. J., Lu Y., Agrawal A., Xu T. (2021). Multifunctional
Metasurfaces Enabled by Simultaneous and Independent
Control of Phase and Amplitude for Orthogonal Polarization States. Light: Sci. Appl..

[26] Zeng Y., Sha X., Zhang C., Zhang Y., Deng H., Lu H., Qu G., Xiao S., Yu S., Kivshar Y., Song Q. (2025). Metalasers
with Arbitrarily Shaped Wavefront. Nature.

[27] Huang H., Overvig A. C., Xu Y., Malek S. C., Tsai C.-C., Alù A., Yu N. (2023). Leaky-Wave Metasurfaces for Integrated
Photonics. Nat. Nanotechnol..

[28] Arbabi A., Horie Y., Bagheri M., Faraon A. (2015). Dielectric Metasurfaces
for Complete Control of Phase and Polarization with Subwavelength
Spatial Resolution and High Transmission. Nat.
Nanotechnol..

[29] Dorrah A. H., Rubin N. A., Zaidi A., Tamagnone M., Capasso F. (2021). Metasurface Optics for on-Demand Polarization Transformations
Along the Optical Path. Nat. Photonics.

[30] Li G., Zhang S., Zentgraf T. (2017). Nonlinear
Photonic Metasurfaces. Nat. Rev. Mater..

[31] Mamin H. J., Rugar D. (1992). Thermomechanical Writing with an
Atomic Force Microscope Tip. Appl. Phys. Lett..

[32] Pires D., Hedrick J. L., De Silva A., Frommer J., Gotsmann B., Wolf H., Despont M., Duerig U., Knoll A. W. (2010). Nanoscale
Three-Dimensional Patterning of Molecular Resists by Scanning Probes. Science.

[33] Garcia R., Knoll A. W., Riedo E. (2014). Advanced Scanning Probe Lithography. Nat. Nanotechnol..

[34] Lassaline N. (2024). Generating
Smooth Potential Landscapes with Thermal Scanning-Probe Lithography. J. Phys. Mater..

[35] Lassaline N., Brechbühler R., Vonk S. J. W., Ridderbeek K., Spieser M., Bisig S., le Feber B., Rabouw F. T., Norris D. J. (2020). Optical Fourier Surfaces. Nature.

[36] Glauser Y. M., Maris J. J. E., Brechbuhler R., Crimmann J. G., De Rosa V. G., Petter D., Nagamine G., Lassaline N., Norris D. J. (2025). Diffraction of Light from Optical
Fourier Surfaces. ACS Photonics.

[37] Glauser Y. M., Vonk S. J. W., Seda D. B., Niese H., de Jong B., Bidaut M. F., Bossavit E., Petter D., Nagamine G., Lassaline N., Norris D. J. (2026). Fourier Pixels for Bidirectional
Light Control. Nature.

[38] Lassaline, N. ; Chelladurai, D. ; Kohli, M. ; Ulrich, R. ; Glauser, Y. ; Petter, D. ; Leuthold, J. ; Norris, D. J. Fourier Surface Grating Couplers for Integrated Photonics. In Optica Advanced Photonics Congress; Optica Publishing Group, 2022. DOI: 10.1364/IPRSN.2022.ITh2B.5

[39] Petter D., Kaufmann F., Chelladurai D., Kohli M., Maeder A., Glauser Y. M., Lassaline N., Maris J. J. E., Leuthold J., Grange R., Norris D. J. (2026). Optical
Fourier Surfaces for Integrated
Photonics. ACS Nano.

[40] Vonk S. J. W., de Jong B., Glauser Y. M., Seda D. B., Bidaut M. F., Savinson B., Niese H., Norris D. J. (2026). Broadband, Compact,
and Training-Free Optical Processors for Parallel Image Classification. ACS Nano.

[41] Gerchberg R. W. (1972). A Practical
Algorithm for the Determination of Plane from Image and Diffraction
Pictures. Optik.

[42] Fienup J. R. (1982). Phase Retrieval
Algorithms: A Comparison. Appl. Opt..

[43] Li J., Gan T., Bai B., Luo Y., Jarrahi M., Ozcan A. (2023). Massively
Parallel Universal Linear Transformations Using a Wavelength-Multiplexed
Diffractive Optical Network. Adv. Photonics.

[44] Luo Y., Mengu D., Yardimci N. T., Rivenson Y., Veli M., Jarrahi M., Ozcan A. (2019). Design of Task-Specific Optical Systems
Using Broadband Diffractive Neural Networks. Light: Sci. Appl..

[45] McPeak K. M., Jayanti S. V., Kress S. J. P., Meyer S., Iotti S., Rossinelli A., Norris D. J. (2015). Plasmonic Films Can Easily Be Better:
Rules and Recipes. ACS Photonics.

[46] Nagpal P., Lindquist N. C., Oh S. H., Norris D. J. (2009). Ultrasmooth Patterned
Metals for Plasmonics and Metamaterials. Science.

[47] Harvey J., Pfisterer R. (2019). Understanding
Diffraction Grating Behavior: Including
Conical Diffraction and Rayleigh Anomalies from Transmission Gratings. Opt. Eng..

[48] Harvey J., Pfisterer R. (2020). Understanding
Diffraction Grating Behavior, Part II:
Parametric Diffraction Efficiency of Sinusoidal Reflection (Holographic)
Gratings. Opt. Eng..

[49] Sui X., He Z., Chu D., Cao L. (2024). Non-Convex Optimization for Inverse
Problem Solving in Computer-Generated Holography. Light: Sci. Appl..

[50] Matsushima K., Shimobaba T. (2009). Band-Limited
Angular Spectrum Method for Numerical
Simulation of Free-Space Propagation in Far and near Fields. Opt. Express.

[51] Vitiello M. S., Nobile M., Ronzani A., Tredicucci A., Castellano F., Talora V., Li L., Linfield E. H., Davies A. G. (2014). Photonic Quasi-Crystal Terahertz Lasers. Nat. Commun..

[52] Zupancic P., Preiss P. M., Ma R., Lukin A., Eric Tai M., Rispoli M., Islam R., Greiner M. (2016). Ultra-Precise Holographic
Beam Shaping for Microscopic Quantum Control. Opt. Express.

[53] Pasienski M., DeMarco B. (2008). A High-Accuracy Algorithm for Designing Arbitrary Holographic
Atom Traps. Opt. Express.

[54] Chang C., Xia J., Yang L., Lei W., Yang Z., Chen J. (2015). Speckle-Suppressed
Phase-Only Holographic Three-Dimensional Display Based on Double-Constraint
Gerchberg–Saxton Algorithm. Appl. Opt..

[55] Fienup J. (1980). Iterative
Method Applied to Image Reconstruction and to Computer-Generated Holograms. Opt. Eng..

[56] Hou Y.-H., Li Z.-S., Zheng Y.-W., Huang Q., Li Y.-L., Wang D., Wang Q.-H. (2025). High-Quality
and Efficient Phase-Only
Hologram Generation Method Based on Complex Amplitude Constrained
Gerchberg-Saxton Algorithm. Displays.

[57] Rumelhart D. E., Hinton G. E., Williams R. J. (1986). Learning
Representations by Back-Propagating
Errors. Nature.

[58] Lassaline N., Thureja D., Chervy T., Petter D., Murthy P. A., Knoll A. W., Norris D. J. (2021). Freeform
Electronic and Photonic
Landscapes in Hexagonal Boron Nitride. Nano
Lett..

[59] Sørensen C. H., Nielsen M. V., Linde S. J., Nguyen D. H., Iversen C. E., Jensen R., Raza S., Bøggild P., Booth T. J., Lassaline N. (2026). Quantitative Mapping of Smooth Topographic
Landscapes Generated Using Thermal Scanning-Probe Lithography. Nat. Protoc..

[60] Cueff S., Berguiga L., Nguyen H. S. (2024). Fourier Imaging
for Nanophotonics. Nanophotonics.

[61] Zhang J., Zhang L., Xu W. (2012). Surface Plasmon
Polaritons: Physics
and Applications. J. Phys. D: Appl. Phys..

[62] Glauser, Y. M. ; Shen, C.-Y. ; Seda, D. B. ; Işil, Ç. ; Benito Eberhard, P. ; Niese, H. ; Crimmann, J. G. ; Petter, D. ; Nagamine, G. ; Vonk, S. J. W. ; Lassaline, N. ; Ozcan, A. ; Norris, D. J. , Optical Fourier Surfaces for Free-Space Computer-Generated Holography: arXiv:2607.09307, 2026. https://arxiv.org/abs/2607.09307. (accessed September 23, 2026). 10.1021/acsnano.6c13095 PMC13738787 42975268

