# Supplementary material for: Optical Fourier Surfaces for Free-Space Computer-Generated Holography
Source: ACS Nano. 2026 Oct 19;20(43):29091–101. doi: 10.1021/acsnano.6c13095 (PMC13738787; doi:10.1021/acsnano.6c13095)
Supplement: Supplementary file 1 [file nn6c13095_si_001.pdf]

Supporting Information for

Optical Fourier Surfaces for Free-Space Computer-Generated Holography

Yannik M. Glauser,[1] *Che-Yung Shen,*[2,3,4] *David B. Seda,*[1] *Çağatay Işil,*[2,3,4] *Patrick Benito Eberhard,*[1] *Hannah Niese,*[1] *Juri G. Crimmann,*[1] *Daniel Petter,*[1] *Gabriel Nagamine,*[1] *Sander J. W. Vonk,*[1] *Nolan Lassaline,*[1,6] *Aydogan Ozcan,*[2,3,4,5] *and David J. Norris*[1,*]

[1]Optical Materials Engineering Laboratory, Department of Mechanical and Process Engineering, ETH Zurich, 8092 Zurich, Switzerland

[2]Department of Electrical and Computer Engineering, University of California, 90095 Los Angeles, USA

[3]Department of Bioengineering, University of California, 90095 Los Angeles, USA

[4]California NanoSystems Institute (CNSI), University of California, 90095 Los Angeles, USA

[5]Department of Surgery, David Geffen School of Medicine, University of California, 90095 Los Angeles, USA

[6]Department of Physics, Technical University of Denmark, 2800 Kongens Lyngby, Denmark

Corresponding author

*Email: dnorris@ethz.ch

S1. SUPPORTING DISCUSSION

S1.1 SCALAR DIFFRACTION THEORY

The following section discusses scalar diffraction theory in the context of Fourier optics and linear system theory. It combines information from refs S1 and S2 with explanations from Goodman[S3] and Saleh and Teich.[S4]

Light can be described as an electromagnetic wave, whose propagation is governed by Maxwell's equations. In the absence of free charges and currents, these equations reduce to a set of equations for all components of the electric field **E** and the magnetic field **H**, which simplifies to a scalar-wave equation:

$$\nabla^2 u(\mathbf{r},t) - \frac{n^2}{c^2}\frac{\partial^2 u(\mathbf{r},t)}{\partial t^2} = 0\,, \tag{S1}$$

where $u(\mathbf{r},t)$ describes any field component as a function of position $\mathbf{r}$ and time t, c is the speed of light in vacuum, and n is the refractive index.

General solutions to eq S1 are monochromatic waves

$$u(\mathbf{r},t) = A(\mathbf{r})\cos[\omega t - \phi(\mathbf{r})] \tag{S2}$$

with amplitude $A(\mathbf{r})$, phase $\phi(\mathbf{r})$, and angular frequency ω. Alternatively, the scalar field can be written as the real part of a complex function $U(\mathbf{r})$,

$$u(\mathbf{r},t) = \mathrm{Re}[U(\mathbf{r})\exp(-i\omega t)] \tag{S3}$$

with

$$U(\mathbf{r}) = A(\mathbf{r})\exp[i\phi(\mathbf{r})]\,. \tag{S4}$$

Inserting eq S4 into the scalar-wave equation eq S1 removes the harmonic time dependence of the monochromatic wave and leads to the Helmholtz equation

$$(\nabla^2 + k^2)U(\mathbf{r}) = 0\,. \tag{S5}$$

The wavenumber k is defined by

$$k = \frac{n\omega}{c} = \frac{2\pi n}{\lambda}, \tag{S6}$$

with wavelength λ of the light in vacuum. Among the solutions of the Helmholtz equation, the simplest set are plane waves

$$U(\mathbf{r}) = \exp(i\mathbf{k} \cdot \mathbf{r}), \tag{S7}$$

which are defined by their wavevector $\mathbf{k} = (k_x, k_y, k_z)$ with $|\mathbf{k}| = k$. For propagation along the z axis, k_z follows from the wavenumber k and the in-plane momenta (k_x, k_y) as

$$k_z = \sqrt{k^2 - {k_x}^2 - {k_y}^2}, \tag{S8}$$

which describes propagating (${k_x}^2 + {k_y}^2 \leq k^2$, real) or evanescent (${k_x}^2 + {k_y}^2 > k^2$, imaginary) waves.

Decomposing light fields into plane waves builds the foundation of Fourier optics. To obtain the spectrum of spatial frequency components $F(k_x, k_y)$ for a field $f(x, y)$, the Fourier transform is applied:

$$F(k_x, k_y) = \mathcal{F}\{f\} = \iint_{-\infty}^{+\infty} f(x, y) \exp\left[-i\left(k_x x + k_y y\right)\right] \, \mathrm{d}x\mathrm{d}y\,. \tag{S9}$$

Here, we use the non-unitary convention based on angular frequencies. The corresponding inverse Fourier transform is defined as

$$f(x, y) = \mathcal{F}^{-1}\{F\} = \frac{1}{4\pi^2} \iint_{-\infty}^{+\infty} F(k_x, k_y) \exp\left[i\left(k_x x + k_y y\right)\right] \, \mathrm{d}k_x\mathrm{d}k_y\,. \tag{S10}$$

Fourier analysis is crucial for characterizing linear systems. It is widely applied to describe light propagation through free space and linear optical components. To explain propagation along the z direction from an input plane ($z = 0$) to an output plane ($z = d$), we consider the field distributions $U(\mathbf{r})$ at the two parallel planes. Assuming a linear system, the output field $U(x, y, d) = f_{\mathrm{out}}(x, y)$ is linearly related to the input field $U(\xi, \eta, 0) = f_{\mathrm{in}}(\xi, \eta)$. According to linear system theory, such a system is fully characterized by its impulse response $h(\xi, \eta, x, y)$. For a linear space-invariant optical

system, the impulse response reduces to $h(x-\xi, y-\eta)$, depending only on relative coordinates. The output field $f_{\mathrm{out}}(x, y)$ can then be written as the convolution integral:

$$f_{\mathrm{out}}(x, y) = \iint\limits_{-\infty}^{+\infty} f_{\mathrm{in}}(\xi, \eta) h(x-\xi, y-\eta)\, \mathrm{d}\xi \mathrm{d}\eta\,. \tag{S11}$$

By applying the convolution theorem of the Fourier transform, a linear space-invariant optical system can equivalently be described by its transfer function $H(k_x, k_y)$, which is the Fourier transform of $h(x-\xi, y-\eta)$. It simply relates the Fourier-transformed input and output signals as a multiplication:

$$F_{\mathrm{out}}(k_x, k_y) \;=\; F_{\mathrm{in}}(k_x, k_y)\, H(k_x, k_y)\,. \tag{S12}$$

For many applications, it is more convenient to perform propagation calculations in Fourier space (eq S12). In this case, the wavefront $f_{\mathrm{in}}(x, y)$ is decomposed into its angular frequency components $F_{\mathrm{in}}(k_x, k_y)$. These components are propagated by multiplication with the transfer function in Fourier space $H(k_x, k_y)$ and then transformed back to real space by the inverse Fourier transform. This approach is known as the angular spectrum method.

For propagation of light along the z axis, the impulse response $h(x-\xi, y-\eta)$, based on the first Rayleigh-Sommerfeld solution, is defined as

$$h(x-\xi, y-\eta) = \frac{1}{2\pi}\frac{d}{r}\left(\frac{1}{r} - jk\right)\frac{\exp(ikr)}{r}\,, \tag{S13}$$

where

$$r = \sqrt{(x-\xi)^2 + (y-\eta)^2 + d^2}\,. \tag{S14}$$

The corresponding transfer function $H(k_x, k_y)$ is given by

$$H(k_x, k_y) = \exp(ik_z d) = \exp\left(i\sqrt{k^2 - k_x^2 - k_y^2}\, d\right), \tag{S15}$$

where we applied the definition of k_z from eq S8. When the distance d between the input and output plane is large compared to the wavelength λ, that is $d \gg \lambda$, the impulse response in eq S13 simplifies to

$$h(x-\xi, y-\eta) = \frac{d}{i\lambda}\frac{\exp(ikr)}{r^2}\,. \tag{S16}$$

Additional approximations can be introduced, leading to the Fresnel and Fraunhofer diffraction regimes. The Fresnel approximation is based on the Taylor expansion of eq S14. Under the assumption that

$$\max\left\{\frac{\pi}{4\lambda}[(x-\xi)^2+(y-\eta)^2]^2\right\} \ll d^3\,, \tag{S17}$$

only the first two orders of the expansion are retained, resulting in

$$r = d\left[1+\frac{1}{2}\left(\frac{x-\xi}{d}\right)^2+\frac{1}{2}\left(\frac{y-\eta}{d}\right)^2\right]. \tag{S18}$$

Substituting eq S18 into eq S16 yields

$$h(x-\xi, y-\eta) = \frac{e^{jkd}}{i\lambda d}\exp\left\{\frac{ik}{2d}[(x-\xi)^2+(y-\eta)^2]\right\}. \tag{S19}$$

The corresponding transfer function in the Fresnel regime is

$$H(k_x,k_y) = e^{ikd}\exp\left[-\frac{i\lambda d}{4\pi}\left(k_x^2+k_y^2\right)\right]. \tag{S20}$$

The full convolution integral of eq S11 can then be expressed for the Fresnel approximation by inserting eq S19:

$$f_{\mathrm{out}}(x,y) = \frac{e^{jkd}}{i\lambda d}\iint_{-\infty}^{+\infty} f_{\mathrm{in}}(\xi,\eta)\exp\left\{\frac{ik}{2d}[(x-\xi)^2+(y-\eta)^2]\right\}\mathrm{d}\xi\mathrm{d}\eta\,, \tag{S21}$$

which is known as the Fresnel diffraction integral. The Fresnel approximation restricts the propagation of light to small angles relative to the z axis, meaning $k_x, k_y \ll k$. Therefore, the Fresnel and paraxial approximations are often considered equivalent, defining the Fresnel diffraction regime.

The Fraunhofer approximation introduces an even stronger condition:

$$\max\left[\frac{k(\xi^2+\eta^2)}{2}\right] \ll d\,. \tag{S22}$$

Applying eq S22 to eq S21 yields

$$f_{\mathrm{out}}(x,y) = \frac{e^{ikd}e^{i\frac{k}{2d}(x^2+y^2)}}{i\lambda d}\iint_{-\infty}^{+\infty} f_{\mathrm{in}}(\xi,\eta)\exp\left[-i\frac{k}{d}(x\xi+y\eta)\right]\mathrm{d}\xi\mathrm{d}\eta\,, \tag{S23}$$

which involves the Fourier transform of the input signal $U(\xi,\eta,0) = f_{\mathrm{in}}(\xi,\eta)$ with an additional phase term. This becomes more apparent by rewriting eq S23 as

$$f_{\mathrm{out}}(x,y)=\frac{e^{ikd}e^{i\frac{k}{2d}(x^2+y^2)}}{i\lambda d}F_{\mathrm{in}}\left(\frac{kx}{d},\frac{ky}{d}\right). \tag{S24}$$

The Fraunhofer regime corresponds to the far field. In this context, the far field corresponds to Fourier space because the wave propagation can be directly described by a Fourier transform.

Similar considerations can be made for more complex linear optical systems. For example, lenses can be integrated as optical elements within such systems. As described in the Methods, we employ a $2f$-system in our optical setup to measure the far-field response of our optical Fourier surface (OFS) holograms. In this configuration, the lens performs the Fourier transform of the input signal $U(\xi,\eta,0)=f_{\mathrm{in}}(\xi,\eta)$ without an additional spatially dependent phase term, requiring only the less restrictive Fresnel approximation.

S1.2 PARAXIAL DIFFRACTION MODEL

Based on scalar diffraction theory, the interaction of an incoming reference wave $U_0(x,y)$ with an OFS hologram in silver (Ag) can be expressed through its complex transparency $t(x,y)$:

$$U(x,y)=U_0(x,y)t(x,y)\,. \tag{S25}$$

$U(x,y)$ defines the wavefront directly after reflection. As a phase-only hologram, the OFS locally modulates the phase of the reference wavefront by introducing an optical path difference (OPD). The transparency reads

$$t(x,y)=e^{i\Delta\phi}=e^{i\frac{2\pi}{\lambda}\mathrm{OPD}}=e^{ik\mathrm{OPD}} \tag{S26}$$

with additional local phase $\Delta\phi$. The OPD directly relates to the height profile $h_{\mathrm{p}}(x,y)$ of the OFS hologram through

$$\mathrm{OPD}=-2h_{\mathrm{p}}(x,y)\,. \tag{S27}$$

Here, we employ a paraxial diffraction model. It assumes that light enters and exits the surface profile along the *z* axis, introducing a factor of 2 in the OPD (in and out). The sign convention is chosen such that a negative surface height corresponds to a positive phase shift (i.e., a longer propagation distance).

S1.3 FUNDAMENTAL BUILDING BLOCKS

Sinusoidal and blazed gratings. We consider sinusoidal gratings as intuitive building blocks for far-field holography. Their height profile is defined by

$$h_{\mathrm{p}}(x,y) = A\sin(gx-\varphi)\,, \tag{S28}$$

with amplitude A, phase φ, and spatial frequency $g = \frac{2\pi}{\Lambda}$ (grating momentum), determined by the period Λ. By applying the Jacobi–Anger expansion, the sinusoidal phase term in the transparency can be approximated by

$$t(x,y) = e^{-i\,2kA\sin(gx-\varphi)} = \sum_{m=-\infty}^{\infty} J_m(-2kA)e^{im(gx-\varphi)}\,, \tag{S29}$$

where J_m is the Bessel function of the first kind of order m. Based on eqs S24 and S29, the far-field response $f_{\mathrm{out}}(x,y)$ of an infinite sinusoidal grating is

$$f_{\mathrm{out}}(x,y) \propto \sum_{m=-\infty}^{\infty} J_m(-2kA)e^{-im\varphi}\,\delta(k_x - mg, k_y), \tag{S30}$$

where $k_x = \frac{kx}{d}$, $k_y = \frac{ky}{d}$ for distance d. The diffraction pattern forms a Dirac delta comb, with individual diffraction spots shifted by g with respect to each other in Fourier space. The amplitude of each spot is determined by the Bessel coefficient J_m, which depends on the diffraction order m, wavenumber k, and amplitude A. The phase of each spot is determined by $-m\varphi$.

Following eq 1 of the main text, the superposition of two sinusoidal gratings with arbitrary in-plane wavevectors, $\mathbf{g}_1 = (g_{1,x}, g_{1,y})$ and $\mathbf{g}_2 = (g_{2,x}, g_{2,y})$, produces a convolution of two delta combs, yielding diffraction spots at positions $m\mathbf{g}_1 + n\mathbf{g}_2$ ($m, n \in \mathbb{Z}$):

$$\begin{aligned}
&\left[\sum_{m=-\infty}^{\infty} \delta\left(k_x - mg_{1,x}, k_y - mg_{1,y}\right)\right] * \left[\sum_{n=-\infty}^{\infty} \delta\left(k_x - ng_{2,x}, k_y - ng_{2,y}\right)\right] \\
&= \sum_{m=-\infty}^{\infty}\sum_{n=-\infty}^{\infty} \delta\left[k_x - \left(mg_{1,x} + ng_{2,x}\right), k_y - \left(mg_{1,y} + ng_{2,y}\right)\right] \\
&= \sum_{m=-\infty}^{\infty}\sum_{n=-\infty}^{\infty} \delta[\mathbf{k} - (m\mathbf{g}_1 + n\mathbf{g}_2)]
\end{aligned} \tag{S31}$$

with $\mathbf{k} = (k_x, k_y)$.This principle is exploited for generating arbitrary diffraction images.

In contrast, a blazed grating is defined by a surface profile

$$h_\mathrm{p}(x, y) = \mathrm{mod}\left(\frac{\lambda}{2\Lambda}x, A\right), \tag{S32}$$

with surface period Λ, wavelength λ, and amplitude A. We apply the modulo operator, which represents division with remainder such that the surface height repeats within the range $[0, A)$. Here, the corrugation axis is along x, but it could be set to any in-plane orientation.

If A is a multiple of half the wavelength, the transparency of the blazed grating reduces to

$$t(x, y) = e^{-i2k\cdot\mathrm{mod}\left(\frac{\lambda}{2\Lambda}x, \frac{\lambda}{2}\right)gx} = e^{-i2\frac{2\pi}{\lambda}\cdot\frac{\lambda}{2\Lambda}x} = e^{-igx}, \tag{S33}$$

where eq S32 was applied in eqs S26 and S27. The grating momentum $g = \frac{2\pi}{\Lambda}$. The far-field diffraction response $f_\mathrm{out}(x, y)$ corresponds to a single diffraction spot

$$f_\mathrm{out}(x, y) \propto \delta(k_x - g, k_y), \tag{S34}$$

with $k_x = \frac{kx}{d}, k_y = \frac{ky}{d}$. The superposition of two arbitrary blazed gratings with $\mathbf{g}_1$ and $\mathbf{g}_2$ simply results in another blazed grating with $\mathbf{g} = \mathbf{g}_1 + \mathbf{g}_2$, still diffracting light into only one spot:

$$\delta(\mathbf{k} - \mathbf{g}_1) * \delta(\mathbf{k} - \mathbf{g}_2) = \delta[\mathbf{k} - (\mathbf{g}_1 + \mathbf{g}_2)] = \delta(\mathbf{k} - \mathbf{g}) \tag{S35}$$

with $\mathbf{k} = (k_x, k_y)$. Therefore, superposing blazed gratings adds no optical functionality, making them unsuitable as building blocks for far-field holograms. It is worth noting that blazed gratings diffract light into a single spot only at the specific wavelength for which A is a multiple of $\lambda/2$. This makes them spectrally selective, whereas sinusoidal gratings exhibit broadband capability.

Sinusoidal and blazed lenses. Analogously, we can use sinusoidal lenses as intuitive building blocks for holograms that generate desired diffraction responses at an image plane located a distance $d = f$ from the sample. In cylindrical coordinates, the surface profile is of the form

$$h_\mathrm{p}(\rho, \theta) = A\sin\left(\frac{\pi}{\lambda f}\rho^2 - \varphi\right) \tag{S36}$$

with amplitude A, focal distance f, wavelength λ and phase φ. Applying the Jacobi–Anger expansion (as in eq S29), it can be shown that a sinusoidal lens has multiple foci at distances $\pm f/n$ with $n \in \mathbb{N}^+$, as experimentally verified in Figure S4c.

In contrast, a blazed Fresnel lens exhibits a surface profile

$$h_{\mathrm{p}}(\rho,\theta) = \mathrm{mod}\left(\frac{1}{4f}\rho^2, A\right). \tag{S37}$$

Similar to the blazed grating, if A is a multiple of half the wavelength, the transparency of a blazed Fresnel lens reads

$$t(\rho,\theta) = e^{-i2k\cdot\mathrm{mod}\left(\frac{1}{4f}\rho^2,\frac{\lambda}{2}\right)gx} = e^{-i2\frac{2\pi}{\lambda}\cdot\frac{1}{4f}\rho^2} = e^{-i\frac{\pi}{\lambda f}\rho^2}. \tag{S38}$$

The lens introduces a parabolic phase modulation with a single focus at distance f, as experimentally shown in Figure S4d. The superposition of blazed Fresnel lenses with varying lateral shifts or focal distances simply results in another lens with a parabolic phase profile, providing no additional functionality. This is in strong contrast to the superposition of sinusoidal lenses, which enables richer control over the optical response, while exhibiting broadband capability. Further details are provided in Moreno *et al.*[S5]

S1.4 SUBWAVELENGTH DIFFRACTIVE FEATURES

To investigate the influence of subwavelength surface features on the diffraction response of a phase-only hologram, we analyzed a double-sinusoidal profile of the form

$$h_{\mathrm{p}}(x,y) = A_1\sin(gx) + A_n\sin(ngx-\varphi), \tag{S39}$$

as illustrated in Figure S5a. The first term represents the fundamental harmonic with amplitude A_1 and spatial frequency $g = \frac{2\pi}{\Lambda}$, where the fundamental surface period is Λ. The second term defines the superposed higher harmonic of order n, with amplitude A_n, spatial frequency ng, and relative phase shift φ. Following the discussion in the Supporting Information of ref S1, the diffraction efficiency of order l of a double-sinusoidal OFS, based on the paraxial model, is defined by

$$\eta_l = \left|\sum_{m=-\infty}^{\infty} J_{l-nm}(-2kA_1)J_m(-2kA_n)e^{-im\varphi}\right|^2, \tag{S40}$$

with the wavenumber of light $k = \frac{2\pi}{\lambda}$. For the discussion in Figure S5, we examine the +1st-order diffraction efficiency. We fix l = +1, λ = 550 nm, Λ = 600 nm, A_1 = 50 nm, and φ = 0, while varying A_n = 0–50 nm for n = 2–5. We also account for the fact that only diffraction orders inside the light

cone contribute to the total diffraction efficiency. Therefore, η_{+1} is additionally normalized by a factor K, representing the efficiency ratio between all diffraction orders and those that propagate to the far-field ($l = -1, 0, +1$).[S1,S6,S7] Although the superposed higher harmonic has a period that is smaller than the wavelength of light ($\frac{\Lambda}{n} < \lambda$), corresponding to non-propagating evanescent waves, it still contributes to all the other orders via the convolution principle discussed above (see eq S40 and Figure S5).

S1.5 LINEAR DESIGN APPROACH

The linear approach is based on the first-order Taylor expansion

$$e^{i\phi} = 1 + i\phi + \mathcal{O}[(i\phi)^2]\,, \tag{S41}$$

where we neglect all higher-order terms of phase ϕ. Here ϕ represents the phase modulation as a function of position, $\phi(x, y)$. Applying the Fourier transform to calculate the far-field response leads to

$$\mathcal{F}\{e^{i\phi}\} \approx \mathcal{F}\{1 + i\phi\} \;=\; 4\pi^2\delta(k_x, k_y) + i\mathcal{F}\{\phi\} \tag{S42}$$

with $\delta(k_x, k_y)$ representing the specular reflection (0th-order diffraction) in the center of Fourier space. The only tunable part of the diffraction response is represented by $\mathcal{F}\{\phi\}$. Because the phase shift ϕ, which is introduced by the surface profile of our OFS hologram, is real-valued, $\mathcal{F}\{\phi\}$ must be Hermitian:

$$\mathcal{F}\{\phi\}(-k_x, -k_y) = \mathcal{F}\{\phi\}^*(k_x, k_y)\,. \tag{S43}$$

We take this symmetry constraint into account during the linear design process, when selecting the target diffraction image.

This linear approach has also been applied for diffractive structures that interact with guided plasmonic and photonic modes. In that case, the additional in-plane momentum of the incident wave was anticipated in the design of the surface profile.[S2]

S1.6 (QUASI)CRYSTALS

Azimuthally symmetric superpositions of sinusoidal profiles lead to crystalline (n = 1, 2, 3) or quasicrystalline structures ($n \geq 4$) of the form

$$h_{\mathrm{p}}(x,y) = \frac{A}{n}\sum_{i=0}^{n-1}\cos\left\{g\left[x\cos\left(\frac{\pi}{n}i\right) + y\sin\left(\frac{\pi}{n}i\right)\right]\right\}, \tag{S44}$$

with fixed amplitude A and spatial frequency g. In the limit of infinitely many sinusoidal components ($n \to \infty$), the surface profile becomes rotationally invariant. In cylindrical coordinates, it can be written as

$$h_{\mathrm{p}}(\rho,\theta) = A\,J_0(g\rho) = \frac{A}{\pi}\int_0^{\pi}\cos[g\rho\sin(\tau)]\,d\tau\,, \tag{S45}$$

where J_0 defines the Bessel function of the first kind and zeroth order. The far-field diffraction response of this profile produces a ring of radius g in Fourier space (see Figure S7j).

S1.7 ITERATIVE FOURIER TRANSFORM ALGORITHM

In this work, we applied a generalized Gerchberg–Saxton (GS) algorithm, schematically illustrated in Figure S13. The method iteratively transitions between the object domain (sample plane) and Fourier domain (image plane, far field), imposing constraints on the wavefronts in both domains. In the following discussion, we describe our iterative approach in the context of the original GS algorithm.[S8] Where more general capabilities are introduced through recent modifications, we refer to the relevant literature.

The iteration procedure starts with an initial guess φ_0 for the phase, which we randomly initialize between 0 and 2π. We consider a reference wave $r(x,y)$ with a uniform amplitude $|r|$ and phase $\varphi_{\mathrm{ref}} = 0$. The amplitude profile is a circular spot, accounting for the fact that we collect light from the OFS holograms using a circular aperture in the collection path of our setup (see Figure S3a). Combining the reference wave with the phase profile φ_0 leads to the initial wavefront in the object domain g_0:

$$g_0 = |f|\,e^{i\varphi_0}\,. \tag{S46}$$

During each iteration n, the algorithm goes through 4 steps (see Figure S13):

- **Step 1.** Calculate the corresponding field in the Fourier domain using the Fourier transform:

$$\mathcal{F}\{g_n\} \longrightarrow G_n(k_x, k_y) = |G_n| e^{i\Phi_n} . \qquad \text{(S47)}$$

The Fourier transform of g_n yields G_n with amplitude and phase, $|G_n|$ and Φ_n, respectively. G_n represents the unconstrained far-field response in iteration n.

- **Step 2.** Apply Fourier domain constraints:

$$|F|\, e^{i\Phi_n} \longrightarrow G_n'(k_x, k_y) = |G_n'| e^{i\Phi_n} . \qquad \text{(S48)}$$

The amplitude of the far-field response is set to the target amplitude image $|F|$, while the phase remains unchanged.

We adapted this step by dividing the Fourier domain into a constrained signal region and an unconstrained noise region.[S9] By allowing amplitude noise in irrelevant areas of the Fourier domain, we provide more design freedom for the OFS hologram to generate the desired diffraction image in the signal region. This approach is referred to as mixed-region amplitude freedom (MRAF). It introduces a weight parameter m between 0 and 1, which we set to m = 0.3 for our designs (see Pasienski and DeMarco[S9] for a detailed discussion).

- **Step 3.** Take the inverse Fourier transform back to the object domain:

$$\mathcal{F}^{-1}\{G_n'\} \longrightarrow g_n'(x, y) = |g_n'| e^{i\varphi_n'} . \qquad \text{(S49)}$$

The inverse Fourier transform of G_n' yields g_n', with amplitude and phase, $|g_n'|$ and φ_n', respectively. g_n' represents the unconstrained wavefront in the object domain after iteration n.

- **Step 4.** Apply amplitude constraints in the object plane:

$$|r| e^{i\varphi_n'} \longrightarrow g_{n+1}(x, y) = |g_{n+1}| e^{i\varphi_{n+1}} . \qquad \text{(S50)}$$

The amplitude of the reference wave is set to the circular reference wave $|r|$, while the phase remains unchanged.

We adapted this step by employing the input–output approach by Fienup.[S10,S11] In this context, steps 1 to 3 are considered as a nonlinear function with input g_n and output g_n'. In each iteration, we look for an input that produces an output that best satisfies the object-domain

constraints. Similar to step 2, this approach uses a weight parameter β between 0 and 1, which we set to β = 0.9 for our designs (see Fienup[S10,S11] for a detailed discussion).

After several iterations ($n \leq N$ with N = 100), the algorithm converges to a final phase map ($\varphi_n \rightarrow \varphi_{\mathrm{final}}$), which is then converted to a surface profile using the paraxial diffraction model (eqs S26 and S27). We address the fabrication constraints by applying a Gaussian filter (standard deviation σ = 60 nm) to φ_{final} to smooth the final profile.

S1.8 GRADIENT-BASED DIFFERENTIABLE INVERSE-DESIGN APPROACH FOR WAVELENGTH MULTIPLEXED HOLOGRAMS

To optimize our wavelength-multiplexed OFS designs, we employed a gradient-based differentiable inverse-design framework with a custom-formulated multi-objective loss function. The diffractive reflective designs were trained using stochastic gradient descent with error backpropagation to minimize a loss function $\mathcal{L}$. The loss function is composed of three sub-terms accounting for image reconstruction accuracy ($\mathcal{L}_{\mathrm{image}}$), fabrication feasibility ($\mathcal{L}_h$), and diffraction efficiency ($\mathcal{L}_{\mathrm{eff}}$).

The primary objective is to ensure high-fidelity image reconstruction across all wavelength channels. This is achieved using a normalized mean-squared error (MSE) loss to penalize the structural difference between the diffractive output intensity $O_w(x,y)$ and the ground truth $O_w^{\mathrm{GT}}(x,y)$ within the field of view (FOV) at each wavelength channel λ_w. The loss $\mathcal{L}_w$ for a specific wavelength is expressed as

$$\mathcal{L}_w = \sum_{x=1}^{N_x}\sum_{y=1}^{N_y}\left|O_w^{\mathrm{GT}}(x,y) - \sigma_w O_w(x,y)\right|^2 . \quad \text{(S51)}$$

In this equation, σ_w is a normalization factor for the diffractive output, defined as

$$\sigma_w = \frac{\sum_{x=1}^{N_x}\sum_{y=1}^{N_y} O_w^{\mathrm{GT}}(x,y)}{\sum_{x=1}^{N_x}\sum_{y=1}^{N_y} O_w(x,y)}, \quad \text{(S52)}$$

where N_x and N_y represent the number of discrete steps along the x and y directions within the output FOV, respectively.

During training, the total loss is computed by averaging the individual losses $\mathcal{L}_w$ over all the N_w wavelength channels, ensuring simultaneous optimization for all channels. The overall loss function is then given by

$$\mathcal{L}_{\text{image}} = \sum_{w=1}^{N_w} \alpha_w \mathcal{L}_w \,, \tag{S53}$$

where α_w represents the dynamic channel balance weight for each $\mathcal{L}_w$, which adjusts the performance balance across different wavelength channels during the optimization.[S12] Initially set to 1 for all wavelengths w, α_w is adaptively updated during each training iteration based on the deviation of each channel loss from the mean loss $\mathcal{L}_{\text{mean}} = \sum_{w=1}^{N_w} \mathcal{L}_w$ calculated across all wavelength channels, i.e.,

$$\alpha_w \leftarrow \max[0.1(\mathcal{L}_w - \mathcal{L}_{\text{mean}}) + \alpha_w,\ 0] \,. \tag{S54}$$

To account for fabrication constraints of the diffractive surface, we added two fabrication-aware terms: thickness variation loss and Gaussian smoothing. First, we introduced a thickness variation loss $\mathcal{L}_h$ that suppresses abrupt changes between adjacent pixels in the resulting surface profile. This helps reduce potential simulation–fabrication mismatch by penalizing variations exceeding a threshold h_{thresh}:

$$\begin{aligned} \mathcal{L}_h = & \sum_{x=1}^{N_x^h - 1} \sum_{y=1}^{N_y^h} \max\left[\left|h_{\text{p}}(x+1, y) - h_{\text{p}}(x, y)\right| - h_{\text{thresh}}, 0\right] \\ & + \sum_{x=1}^{N_x^h} \sum_{y=1}^{N_y^h - 1} \max\left[\left|h_{\text{p}}(x, y+1) - h_{\text{p}}(x, y)\right| - h_{\text{thresh}}, 0\right] , \end{aligned} \tag{S55}$$

where N_x^h and N_y^h represent the number of discrete steps along the x and y directions for the diffractive surface, respectively.

In addition, we apply Gaussian smoothing to the diffractive surface to further enhance its spatial smoothness and reduce high-frequency variations that could hinder fabrication. This was implemented by convolving the surface height map $h_{\text{p}}(x, y)$ with a Gaussian kernel $G(x, y)$:

$$h_{\text{smoothed}}(x, y) = \left(h_{\text{p}} * G\right)(x, y) \,. \tag{S56}$$

$G(x, y)$ is the 2D Gaussian kernel, defined as

$$G(x, y) = \frac{1}{2\pi\sigma^2} \exp\left(-\frac{x^2 + y^2}{2\sigma^2}\right), \tag{S57}$$

where σ = 320 nm was used in our designs.

To enhance the energy efficiency of our system, we also introduced a modified loss function by adding a diffraction efficiency penalty term $\mathcal{L}_{\text{eff}}$:

$$\mathcal{L}_{\text{eff}} = \eta_{\text{thresh}} - \min(\eta, \eta_{\text{thresh}}), \tag{S58}$$

$$\eta = \frac{\sum_{w=1}^{N_w} \sum_{x=1}^{N_x} \sum_{y=1}^{N_y} O_w(x, y)}{\sum_{w=1}^{N_w} \sum_{x=1}^{N_x} \sum_{y=1}^{N_y} I_w(x, y)}. \tag{S59}$$

$I_w(x, y)$ is the input intensity at the reflective diffractive surface, and $O_w(x, y)$ is the intensity at the output plane within the FOV at each wavelength channel λ_w. η_{thresh} is the energy efficiency threshold set to maintain a desired output diffraction efficiency. To be specific, when the actual efficiency η falls below η_{thresh}, the efficiency loss becomes non-zero, providing the necessary gradients for the model during training.

Combining all the loss components, the total loss function used for training is

$$\mathcal{L} = \mathcal{L}_{\text{image}} + \beta_h \mathcal{L}_h + \beta_{\text{eff}} \mathcal{L}_{\text{eff}}. \tag{S60}$$

For all the wavelength-multiplexed reflective surface designs reported in the main text, the values of β_{eff} and η_{thresh} were empirically set as 10 and 0.05, respectively. The values of β_h and h_{thresh} were empirically set as 100 and 40 nm, respectively.

We utilized the peak signal-to-noise ratio (PSNR) metric to evaluate the structural fidelity of the intensity image $O_w(x, y)$ produced by the diffractive structure against the ground truth $O_w^{\text{GT}}(x, y)$. For a specific wavelength channel λ_w, the PSNR value is computed using

$$\text{PSNR}_w = -10\log_{10}\left[\sum_x \sum_y \left|O_w^{\text{GT}}(x, y) - \sigma_w O_w(x, y)\right|^2\right]. \tag{S61}$$

The wavelength-multiplexed holograms were designed using a gradient-based differentiable inverse-design framework implemented in Python (version 3.10.4) and JAX (version 0.4.1). A latent height parameter $\theta(x, y)$ was treated as the optimization variable and mapped onto the physical surface-height profile through a sinusoidal parameter, $h(x, y) = \left(\frac{h_{\max}}{2}\right)\left\{\sin\left(\theta(x, y)\right) + 1\right\}$, with $h_{\max}$ = 350 nm. The latent profile was initialized to a uniform zero value, corresponding to a uniform thickness of 175 nm, and was optimized using the AdamW optimizer with an initial learning rate of 0.05. The learning rate was halved every 10 epochs according to a step-decay schedule over 80 epochs (256 iterations per epoch; 20,480 iterations in total). During each iteration, the thickness was constrained to the physically accessible range of [0–350 nm] by the sinusoidal parameter and subsequent clipping, quantized to 256 height levels, and Gaussian-smoothed with σ = 320 nm. Calculations were performed using an NVIDIA GeForce RTX 3090 GPU, an Intel Core i9-11900 CPU and 128 GB of RAM, requiring ~24 mins for a representative 4-channel hologram. The implementation relies on standard, publicly available JAX libraries and functions (https://docs.jax.dev/).

S2. SUPPORTING FIGURES

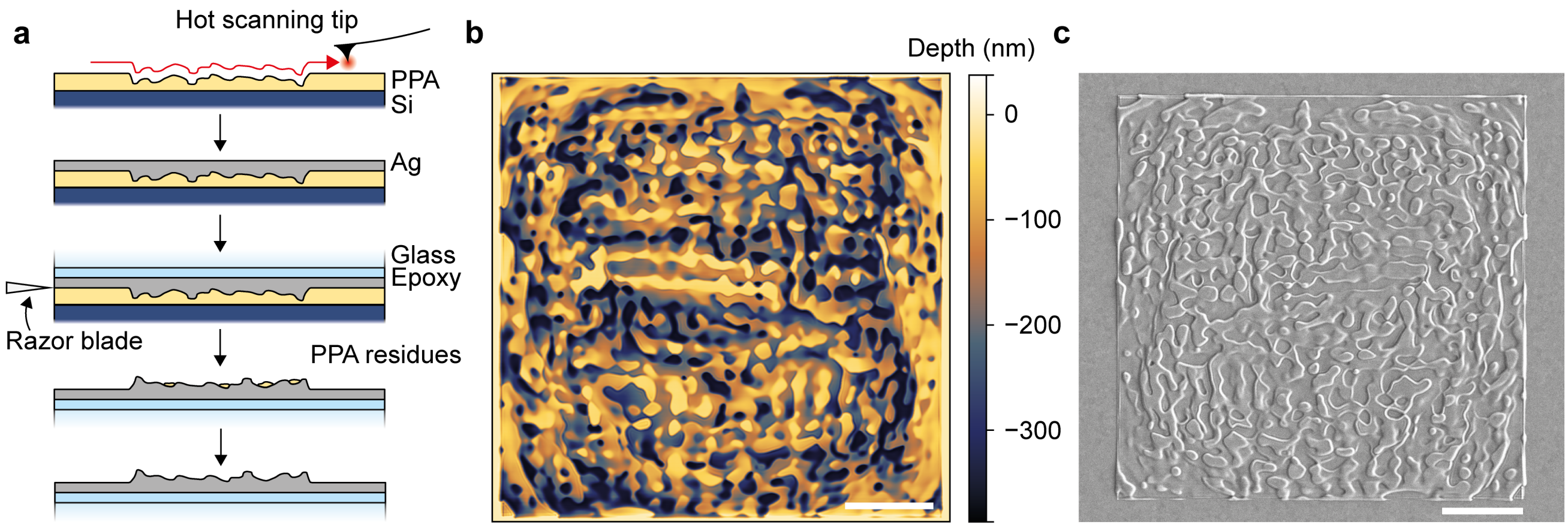

Figure S1. Fabrication procedure of optical Fourier surfaces (OFSs). (a) Process flow for the fabrication of an OFS, incorporating (i) thermal scanning-probe lithography (TSPL), (ii) thermal evaporation of silver (Ag), (iii) template stripping, and (iv) anisole cleaning. (b) Topography data of an OFS hologram (3-fold wavelength-multiplexed, used in Figure 4 in the main text) in poly(phthalaldehyde) (PPA) measured by the TSPL tool. (c) Scanning electron microscopy (SEM) image of the hologram transferred to Ag. The final OFS in Ag is inverted and horizontally mirrored with respect to the structure in PPA due to template stripping. The scale bar in (b) and (c) is 10 μm.

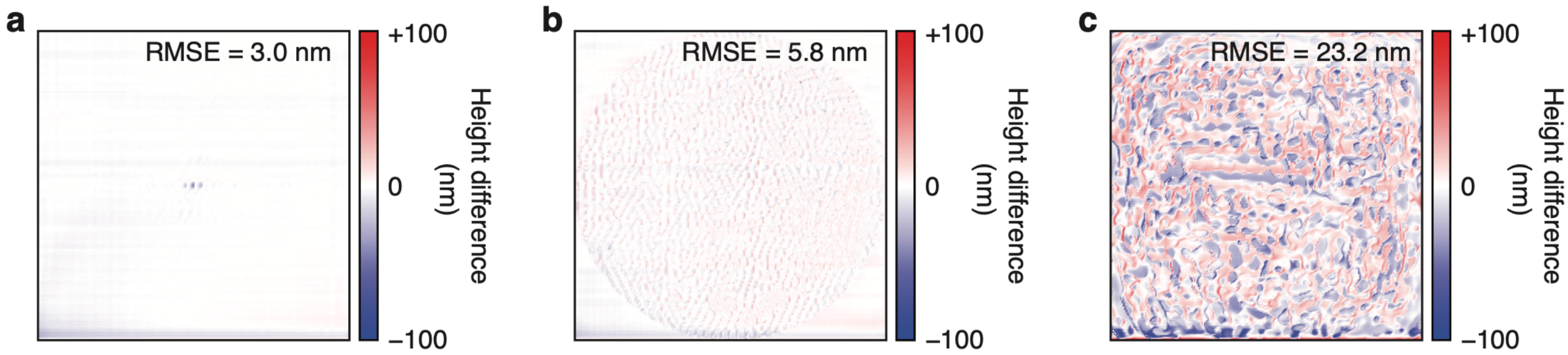

Figure S2. Topography characterization of the different design strategies. (a) Height-difference map between the design and topography in PPA, with the root-mean-squared error (RMSE) value of 3.0 nm. (b,c) Same as (a), but for the Gerchberg–Saxton and gradient-based design approaches. All RMSE values are much smaller than the wavelength of visible light.

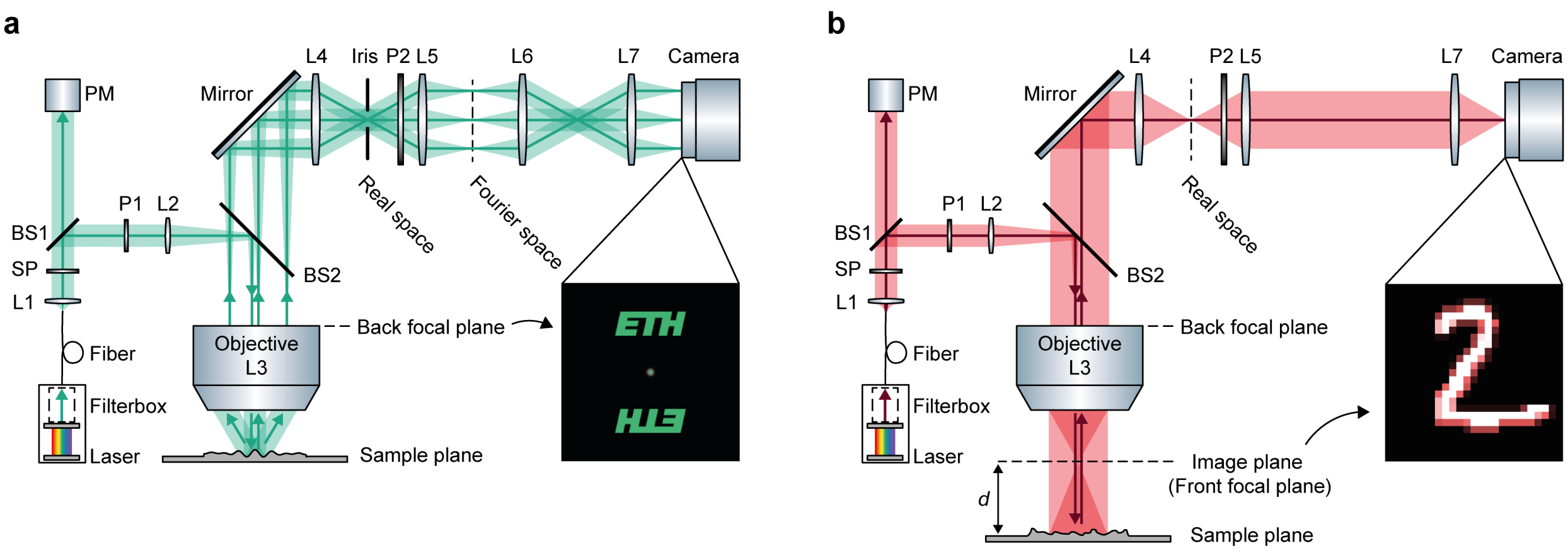

Figure S3. Optical setup with two configurations. (a) Far-field configuration used to image the diffraction response in the back focal plane (Fourier plane) of the objective. For these experiments, the wavelength was fixed at 532 nm. (b) Real-space configuration used to detect the diffraction response in an arbitrary image plane located at a defined distance from the sample plane. For the experiments on spectral multiplexing, the wavelength was swept accordingly. To switch from configuration (a) to (b), lens L6 and the iris were removed, and the sample plane was axially defocused by the desired distance d. The individual optical components of the setup are described in more detail in Methods. Logo used with permission from ETH Zurich.

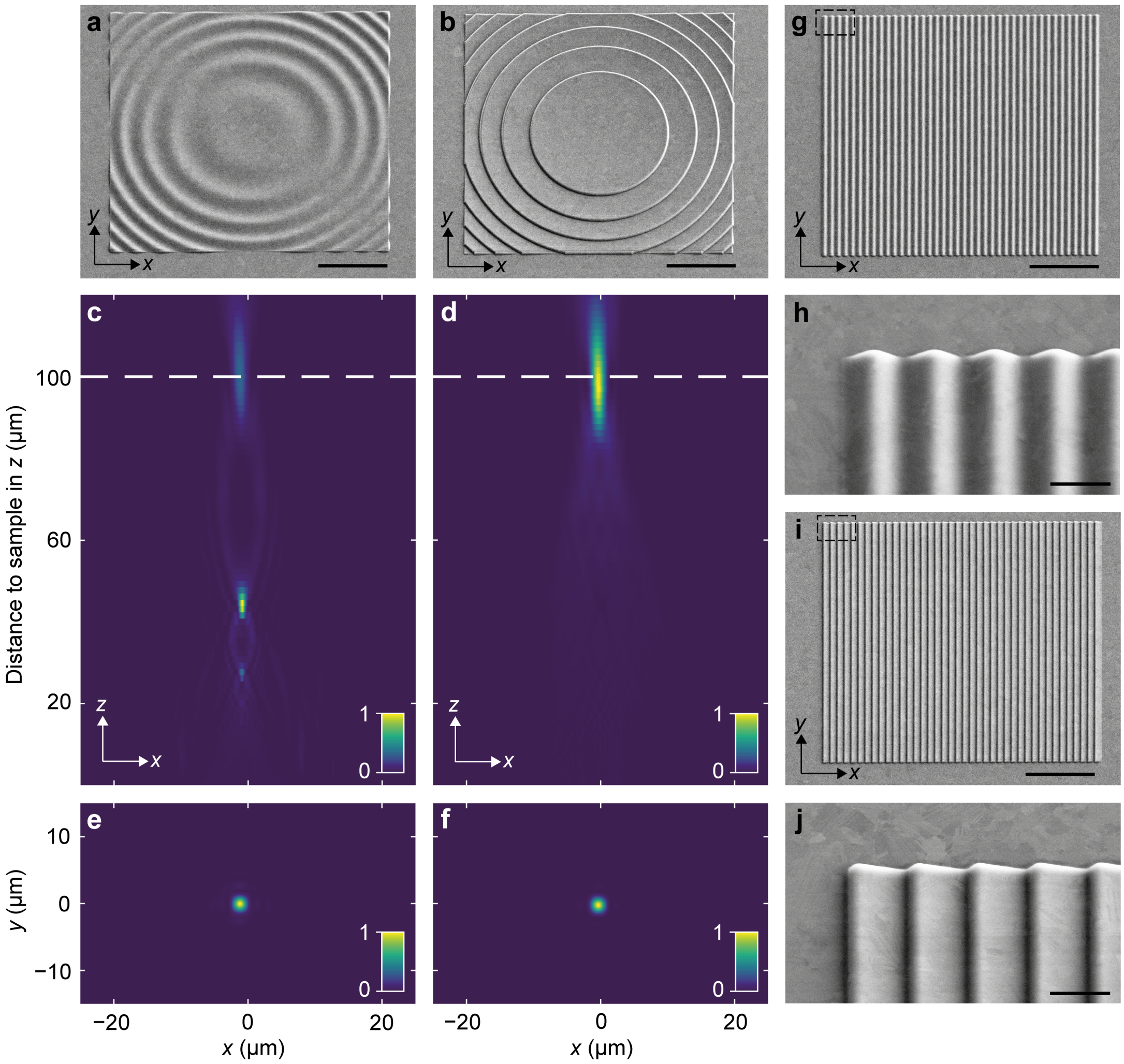

Figure S4. Fundamental structures for holography in the far field and arbitrary image plane. (a),(b) SEM images of a sinusoidal lens and a blazed Fresnel lens in Ag, both with focal length f = 100 µm, depth of 266 nm, and design wavelength λ = 532 nm. (c),(d) Measured intensity profiles as a function of defocus z, showing multiple focal spots for the sinusoidal lens and a single spot at f for the blazed lens, consistent with scalar diffraction theory (see Section S1.3). (e),(f) Cross-sections of (c) and (d) at z = 100 µm (white dashed lines), normalized to their respective maxima. (g),(i) SEM images of sinusoidal and blazed gratings in Ag with depths of 200 and 218 nm and period Λ = 1000 nm. (h),(j) Zoom-ins of the dashed rectangles in (g) and (i). All SEM images were captured at a tilt angle of 30°. Scale bars are 10 µm.

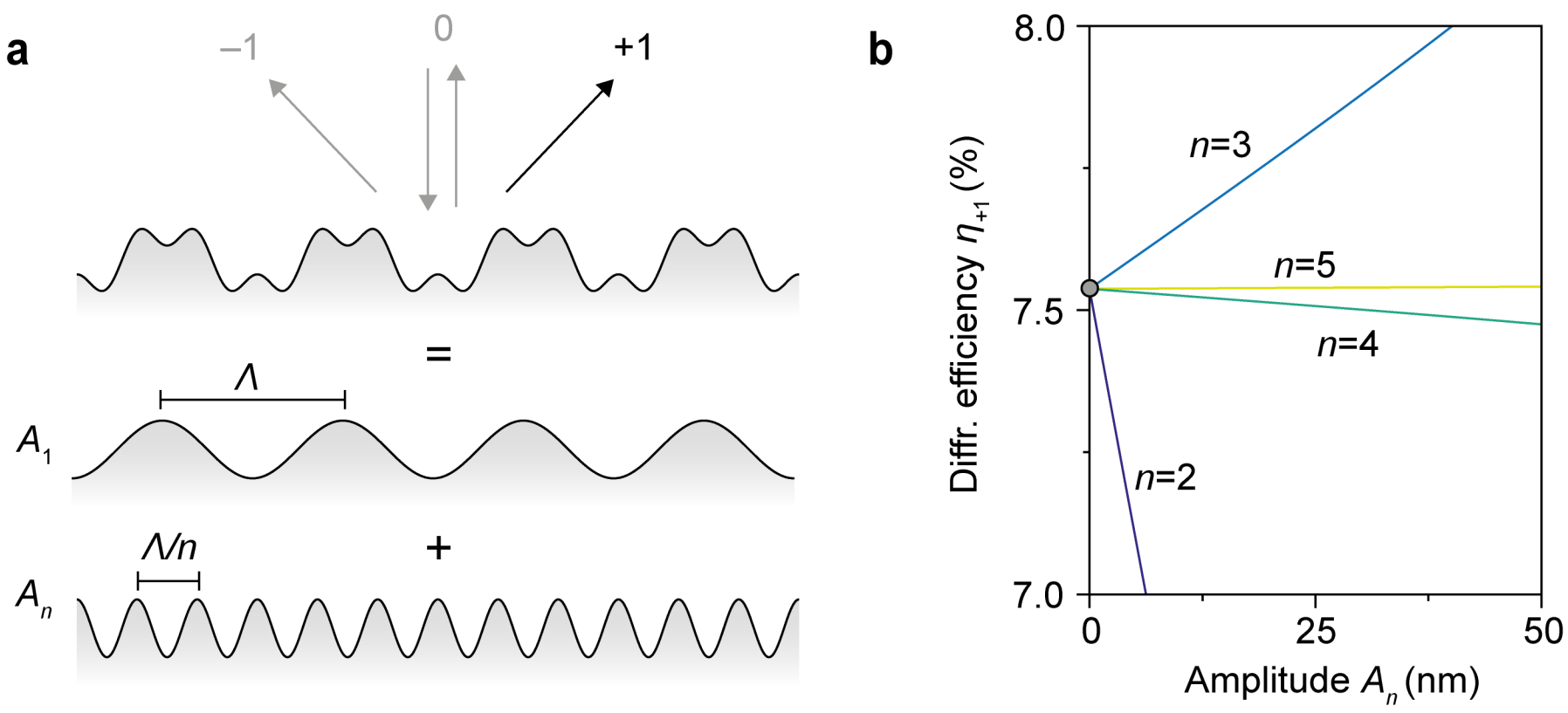

Figure S5. Influence of high spatial frequencies with subwavelength features on the diffraction response of OFSs. (a) Schematic of an OFS composed of a fundamental harmonic with period Λ = 600 nm superposed with a higher harmonic of period Λ/n ($n \geq 2$; $n = 3$ shown here). The amplitude of the fundamental harmonic is fixed at A_1 = 50 nm, while the higher harmonic has a variable amplitude A_n = 0–50 nm (with $A_3 = A_1$ shown here). The influence of the higher-order component on the +1st diffraction order of the OFS is evaluated. The diffraction efficiency is calculated using scalar diffraction theory (see Section S1.4), assuming a wavelength of λ = 550 nm under normal incidence to ensure that only the 0th, +1st, and −1st diffraction orders are non-evanescent. (b) +1st-order diffraction efficiency η_{+1} as a function of higher-harmonic amplitude A_n for n = 2–5. η_{+1} for the fundamental component alone is indicated by the gray circle. The second harmonic (n = 2, purple) significantly modifies the optical response, even though this period does not directly diffract light into the 1st orders. Higher harmonics with n = 3 (blue) and n = 4 (green) induce only minor changes, while harmonics with n = 5 (yellow) and above have a negligible effect, regardless of their amplitude.

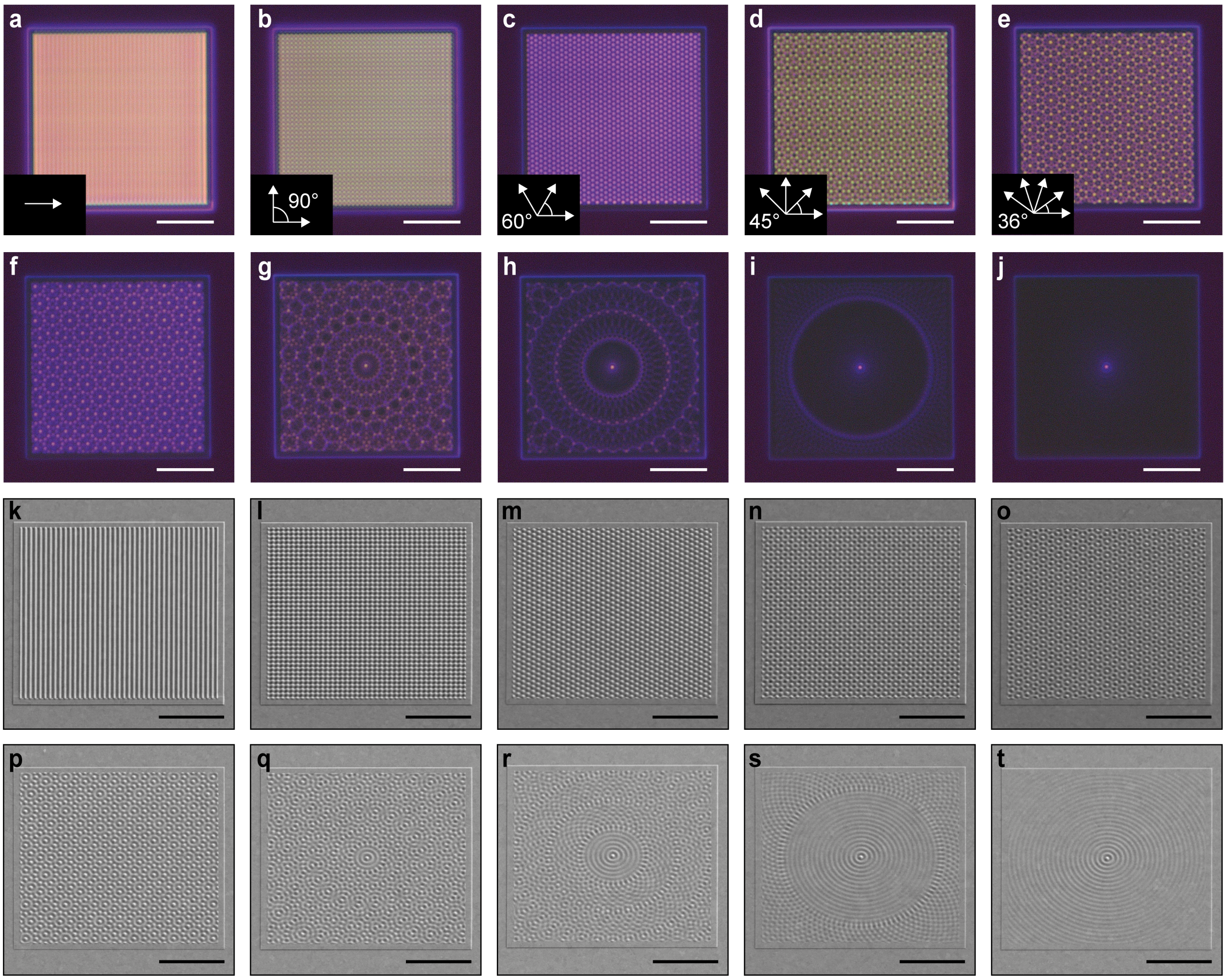

Figure S6. (Quasi)crystalline profiles in PPA and Ag. (a)–(j) Dark-field (DF) images of (quasi)crystalline structures in PPA composed of 1, 2, 3, 4, 5, 6, 10, 20, 50, and infinitely many symmetrically superposed sinusoids with a period of Λ = 750 nm, respectively. The total depth is fixed at 200 nm. In the limit of infinitely many superposed sinusoids, the surface structure becomes rotationally symmetric, with a height profile described by a Bessel function J_0. (k)–(t) SEM images of the corresponding structures transferred to Ag. All SEM images were captured at a tilt angle of 30°. Scale bars in all subpanels are 10 µm.

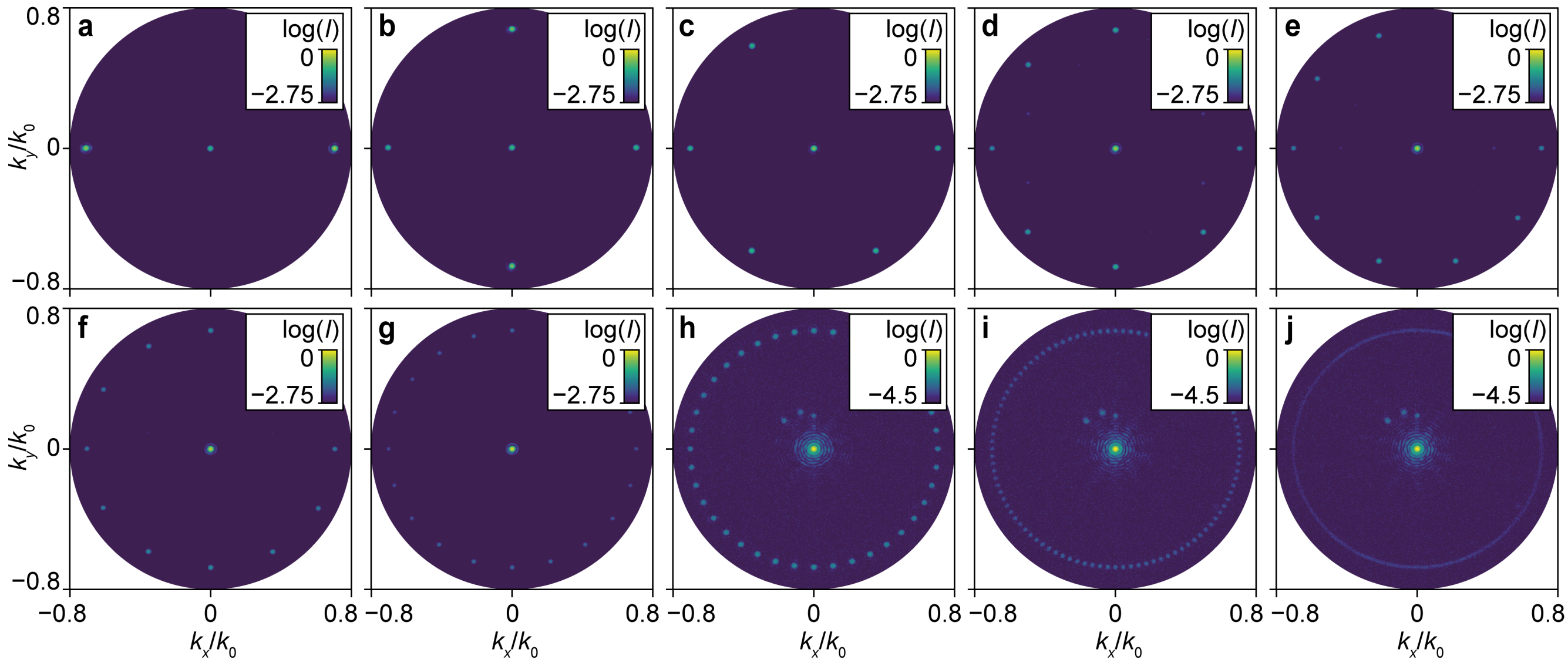

Figure S7. Diffraction patterns of (quasi)crystalline OFSs. (a)–(j) Measured intensity of diffraction in Fourier space (back focal plane of the objective with NA = 0.8) for the (quasi)crystalline structures shown in Figure S6k–t. Coherent light with a wavelength of λ = 532 nm, in the form of a plane wave (i.e., a broad Gaussian beam), was used under normal incidence. The intensities are background-corrected and normalized with respect to the maximum diffraction signal of a flat Ag surface. Data are plotted on a logarithmic intensity scale. Since the intensity of the diffraction spots decreases with increasing numbers of superposed sinusoids, the color scale is adjusted in panels (f)–(j) to enhance visibility. Artifacts around the central 0th-order diffraction spot arise from back reflections within the optical setup.

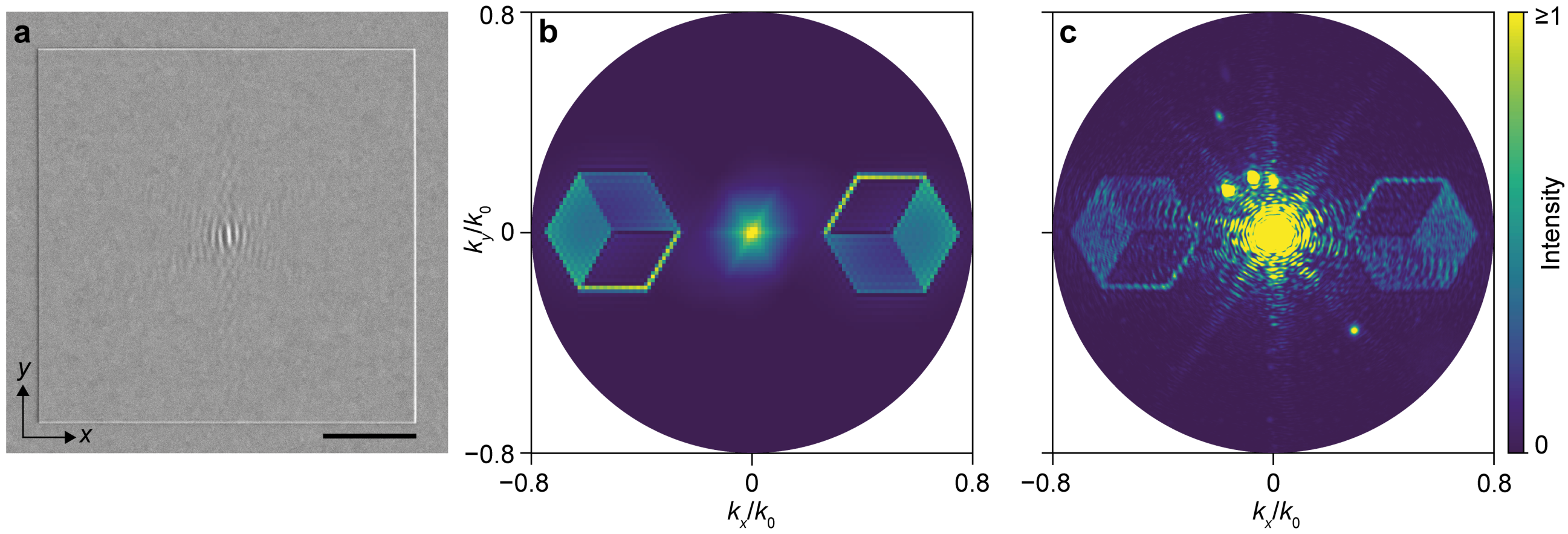

Figure S8. Grayscale diffraction image based on the linear design approach. (a) SEM image of an OFS hologram in Ag, designed to diffract light in the form of a cube with facets of varying intensities. The surface structure has a target depth of 266 nm. The scale bar is 10 µm. (b) Simulated and (c) experimentally measured far-field diffraction intensities in Fourier space (i.e., the back-focal plane of the objective with a numerical aperture NA of 0.8). Intensities in (b) and (c) are normalized to the respective maximum values of the diffraction image (excluding the 0th-order specular reflection) and plotted on a linear scale. The simulation is based on scalar diffraction theory. The experiment was performed under normal incidence using light with λ = 532 nm. Panels (b) and (c) correspond to the grayscale counterpart of Figure 2c,d in the main text.

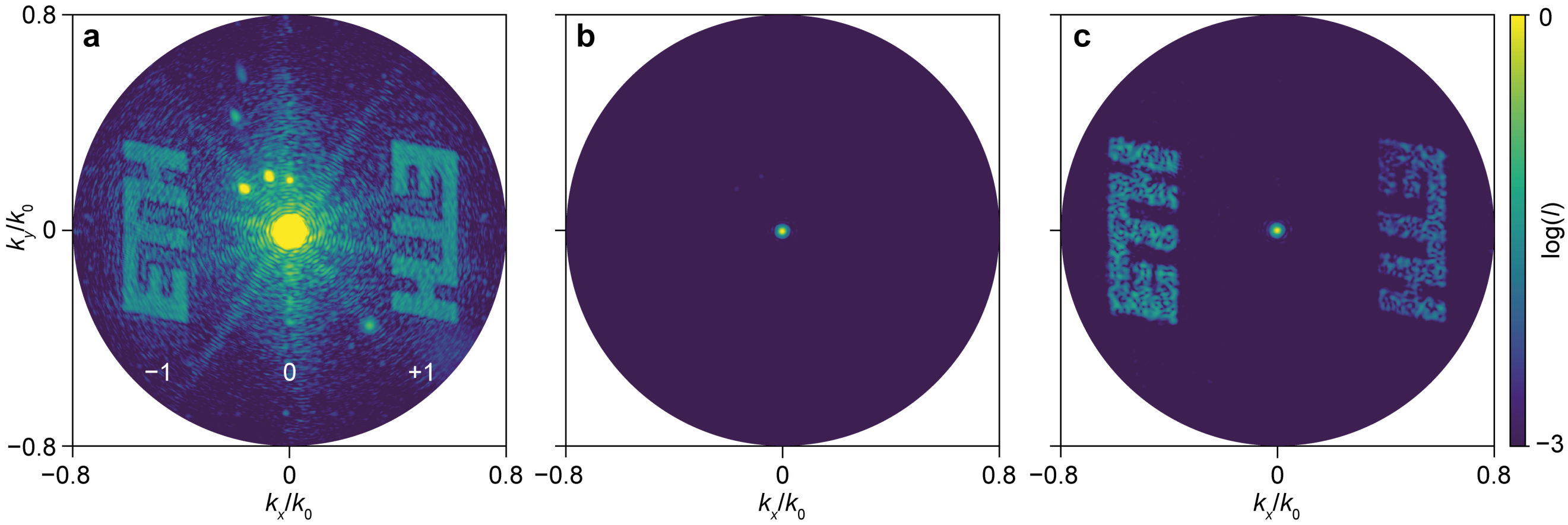

Figure S9. Efficiency characterization for the linear design and iterative Fourier transform algorithm (IFTA). (a) Diffraction image in the back-focal plane from an OFS hologram using the linear approach (intensity data from Figure 2d in the main text, plotted on a log scale). The ETH logo appears symmetrically as the $+1^{st}$ and -1st diffraction orders around the specular reflection (0th order). We saturate the image to clearly observe the diffraction image above the background. (b) Reference measurement on a flat Ag surface showing only the specular reflection. To extract the diffraction efficiency of the OFS hologram in (a), we integrated over the diffraction orders and used the total intensity, corrected for the different acquisition settings, from the reference measurement. We find diffraction efficiencies $\eta_{-1}^{L} = 0.11\%$ and $\eta_{+1}^{L} = 0.10\%$. (c) Same as in (a) but for a hologram designed through the generalized Gerchberg–Saxton (GS) algorithm. Here, we simply integrate over the diffraction orders to find the diffraction efficiencies $\eta_{-1}^{GS} = 32.9\%$ and $\eta_{+1}^{GS} = 15.2\%$. Logo used with permission from ETH Zurich.

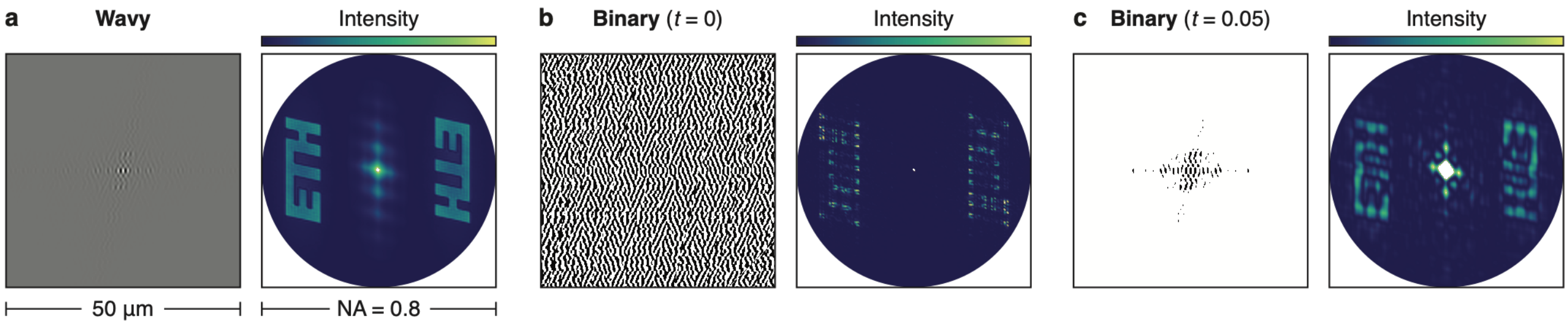

Figure S10. Wavy versus binarized holograms. (a) Linear OFS design used for the hologram (left) in Figure 2 and its simulated optical response (right). (b) Corresponding binarized design and simulated optical response, obtained using a threshold of $t = 0$, separating positive and negative values of the normalized surface profile (between -1 to $+1$). This binarization strongly degrades the reconstructed holographic image. (c) Same as (b), but using a threshold of $t = 0.05$, which retains more of the original holographic response, although still with reduced image quality compared with the wavy design. Logo used with permission from ETH Zurich.

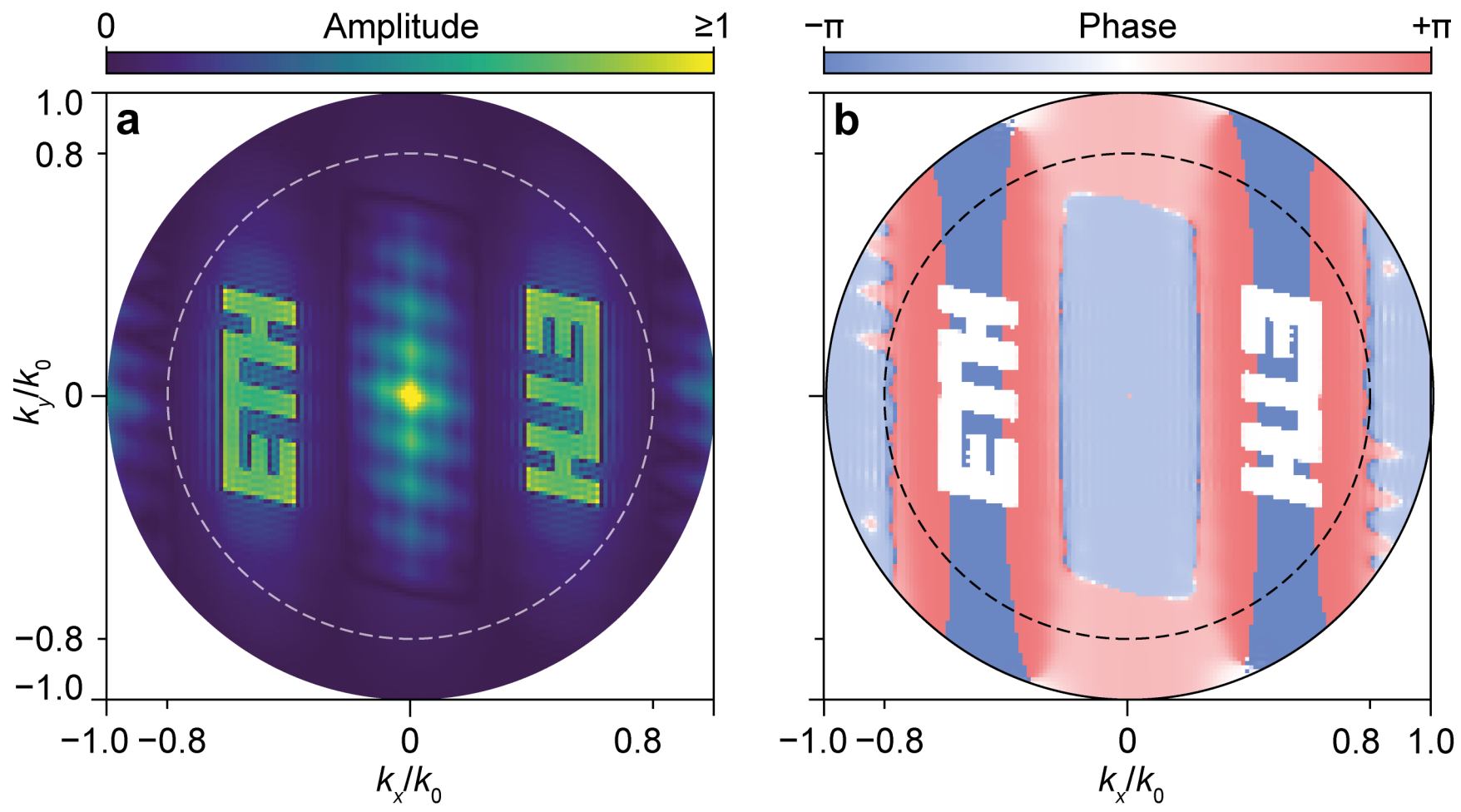

Figure S11. Amplitude and phase control with the linear design approach. (a) Simulated amplitude and (b) simulated phase of the complex wavefront in Fourier space for the OFS hologram depicted in Figure 2a. The numerical aperture NA = 0.8 is indicated by the dashed circle. Simulations are based on scalar diffraction theory, assuming light under normal incidence with $\lambda = 532$ nm. The amplitude in (a) is normalized to the maximum value of the diffraction image (excluding the 0th-order specular reflection) and plotted on a linear scale. The phase in (b) is uniform across the diffraction image, as intended by the design algorithm. Provided that the OFS design constraints are satisfied, in principle, any arbitrary phase map can be realized in the far-field diffraction pattern by appropriately defining the initial complex-valued target image (as shown in Figure 2a). Interestingly, a relative phase shift of approximately π is observed between the diffraction image and background, although the phase of the background in the target image is undefined due to its zero amplitude. This phase shift introduces a node in amplitude between the diffraction image and background, facilitating sharp contours. Logo used with permission from ETH Zurich.

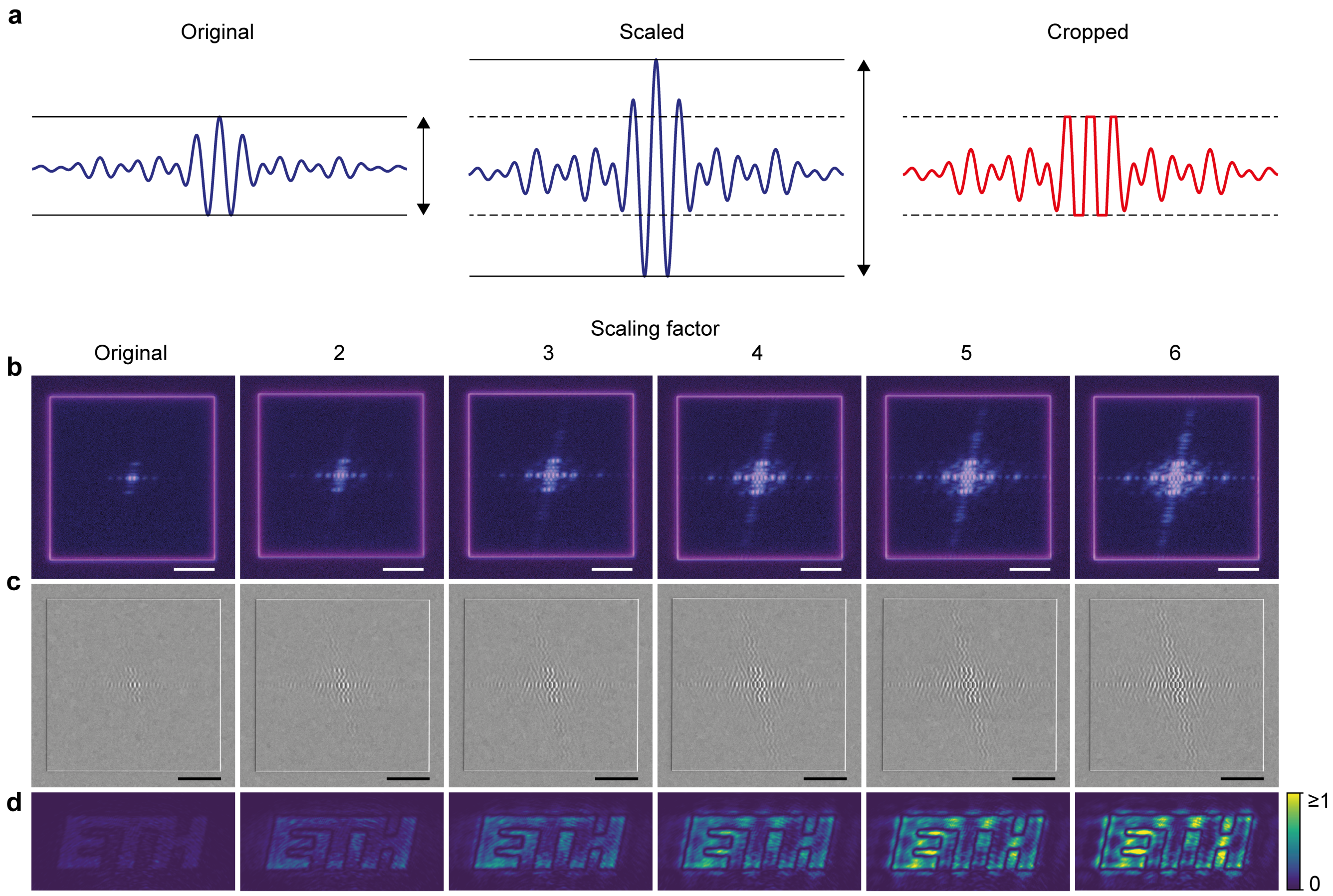

Figure S12. Linear design approach with additional scaling and cropping of the height profile. (a) Strategy to increase the diffracted intensity of the holograms based on the linear design approach. Their original profile (see Figure 2 in the main text) is scaled to increase the contribution of each sinusoidal component. Due to fabrication constraints, the depth of an OFS is limited. We therefore investigate how the diffraction signal is affected when the overall profile is linearly expanded in the *z* direction and cropped to maintain a fixed depth of 266 nm. In the linear design approach, the full depth range is typically used only in the center of the structure, which occupies a small portion of the total area, while most other regions remain shallow. (b) DF images of the scaled and cropped OFS holograms in PPA. The scaling factors range from 1–6, respectively. With increasing scaling factor, the structure becomes more binary in the center, which leads to more scattered light. (c) SEM images of the corresponding OFS holograms in Ag, fabricated from the structures in (b). (d) Diffracted far-field intensity pattern for the structures in (c). Intensities in (d) are normalized to the maximum value of the diffraction image with a scaling factor of 4 (excluding the 0th-order specular reflection) and plotted on a linear scale. The experiment was performed under normal incidence using light with λ = 532 nm. Scale bars in (b) and (c) are 10 µm. Logo used with permission from ETH Zurich.

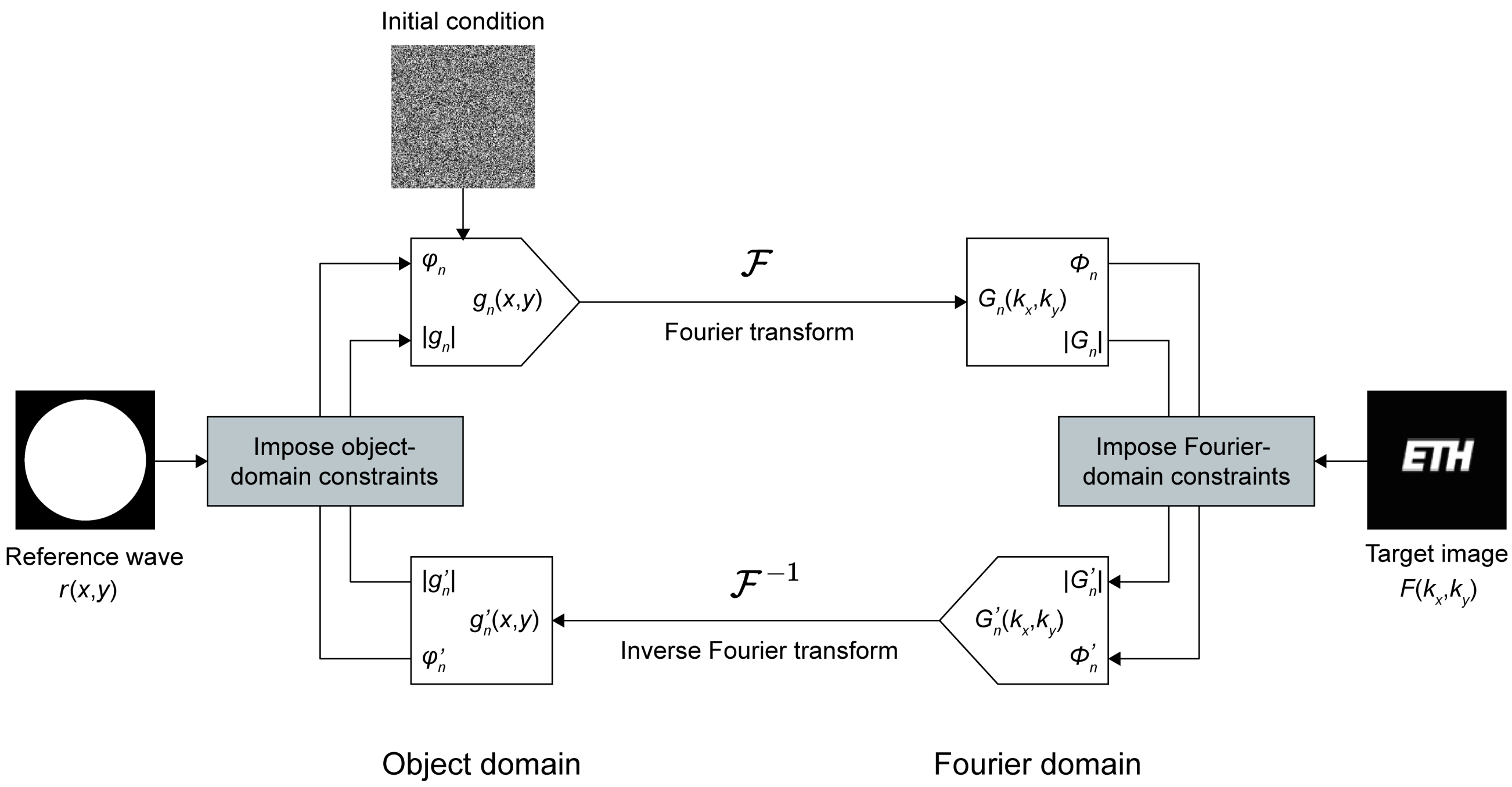

Figure S13. Schematic of the generalized GS algorithm. Each iteration of the algorithm consists of four steps (see Section S1.7 for details). Steps 1 and 3 perform the forward and inverse Fourier transform, respectively, to transition between the object and Fourier domains. In both domains, the optical fields are represented by their amplitude and phase. Step 2 imposes the Fourier-domain constraints by applying the target diffraction image $F(k_x, k_y)$. Step 4 enforces the object-domain constraints, which typically include information from the reference wave $r(x, y)$ or fabrication limitations. Logo used with permission from ETH Zurich.

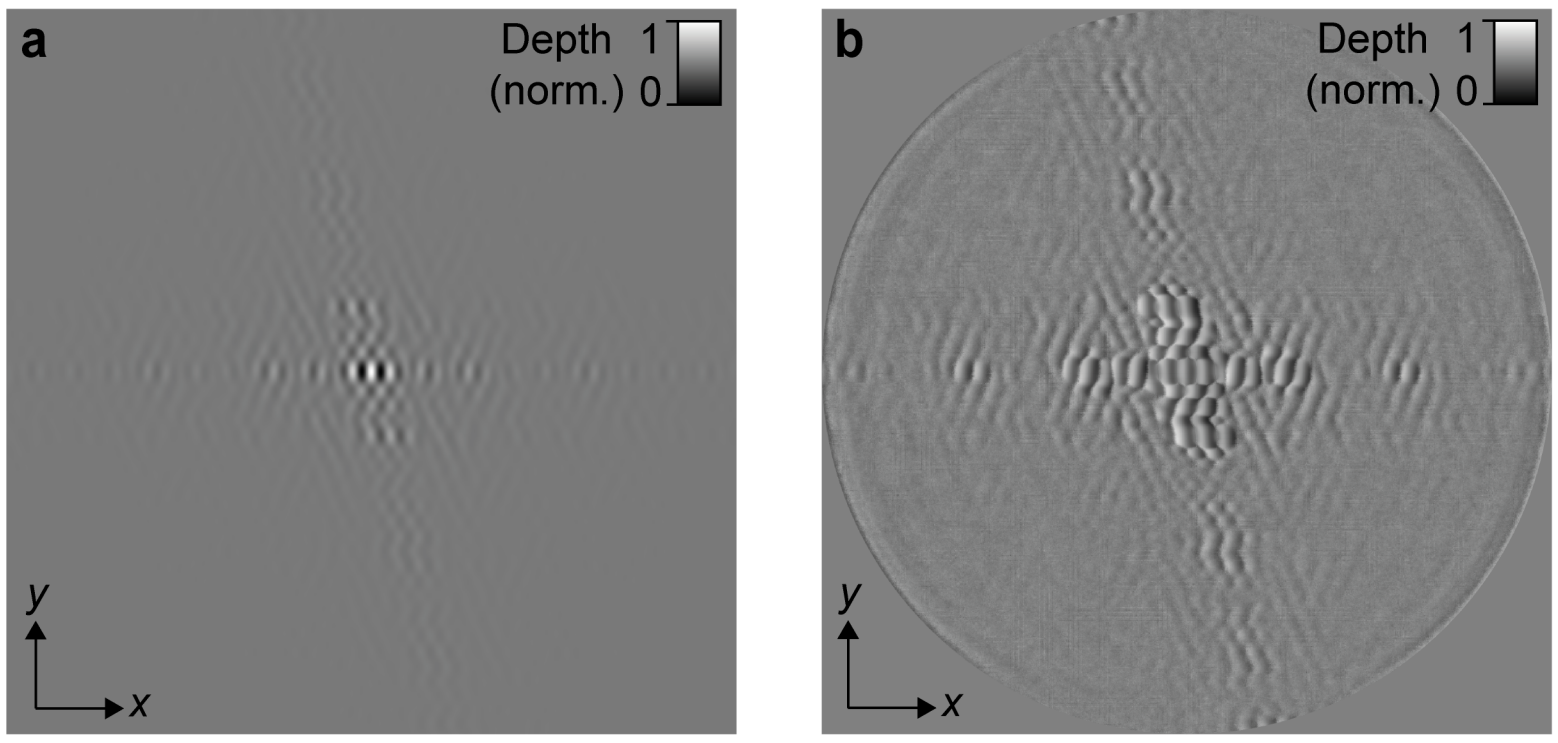

Figure S14. IFTA with additional phase constraints in the Fourier domain. (a) Grayscale design generated using the linear design approach for the ETH logo (see Figure 2 in the main text). (b) Corresponding design obtained with a modified GS algorithm that imposes both amplitude and phase constraints in the Fourier domain to suppress speckles in the diffraction image. The algorithm follows the method of Chang *et al.*,[S13] where we set the parameters (α, β, γ) = (0.2, 0.5, 1.0). The target image mask consisted of a single ETH logo (without its mirror image) with a uniform phase φ = 0, while a random phase distribution was used as the initial condition.

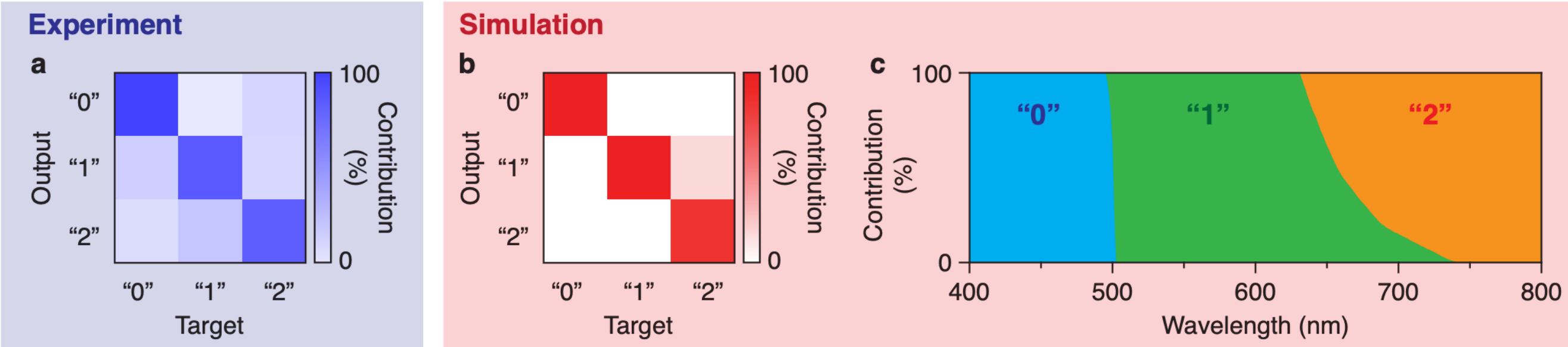

Figure S15. Crosstalk and detuning tolerance of wavelength-multiplexed holograms. (a) Experimental crosstalk matrix obtained by fitting the measured optical response at each illumination wavelength as a linear superposition of the three target images ("0", "1", and "2") and a spatially uniform background. (b) Same as (a), but obtained from the simulated optical responses. (c) Simulated normalized relative contributions of the three target images as a function of illumination wavelength, illustrating the wavelength dependence and spectral selectivity of the multiplexed hologram.

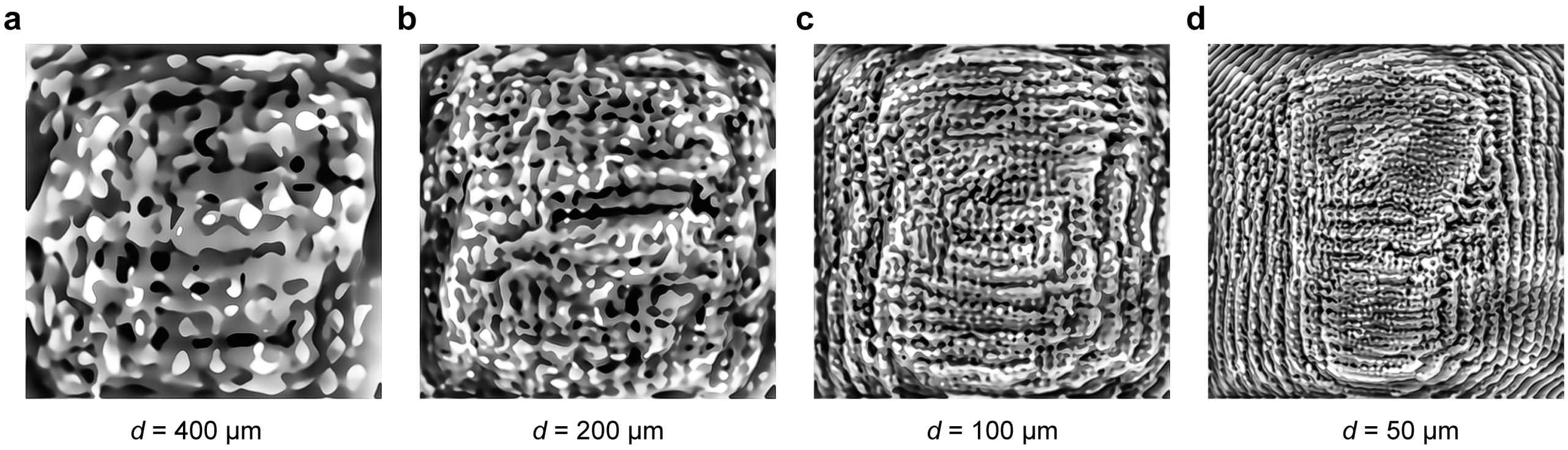

Figure S16. Feature size as a function of image-plane distance. (a)–(d) Grayscale designs of wavelength-multiplexed OFS holograms designed to project images to planes located at distances d = 400, 200, 100, and 50 μm away from the sample plane, respectively. The diffraction images at wavelengths 450, 575, and 700 nm (N_w = 3) correspond to handwritten digits 0, 1, and 2 from the Modified National Institute of Standards and Technology (MNIST) dataset (see Figure 4 in the main text). The lateral hologram size was fixed at 50 μm × 50 μm with a pixel size of 40 nm × 40 nm (1250 × 1250 pixels, as in Figure 4). Grayscale depth values range from 0 to 255 (linear scale), representing a total physical depth of 350 nm. We observe that the structural feature size generally grows as distance d increases, consistent with observations based on diffractive lenses with increasing focal distance ($d = f$, see Section S1.3 for details). Intuitively, stronger light bending requires finer structural features.

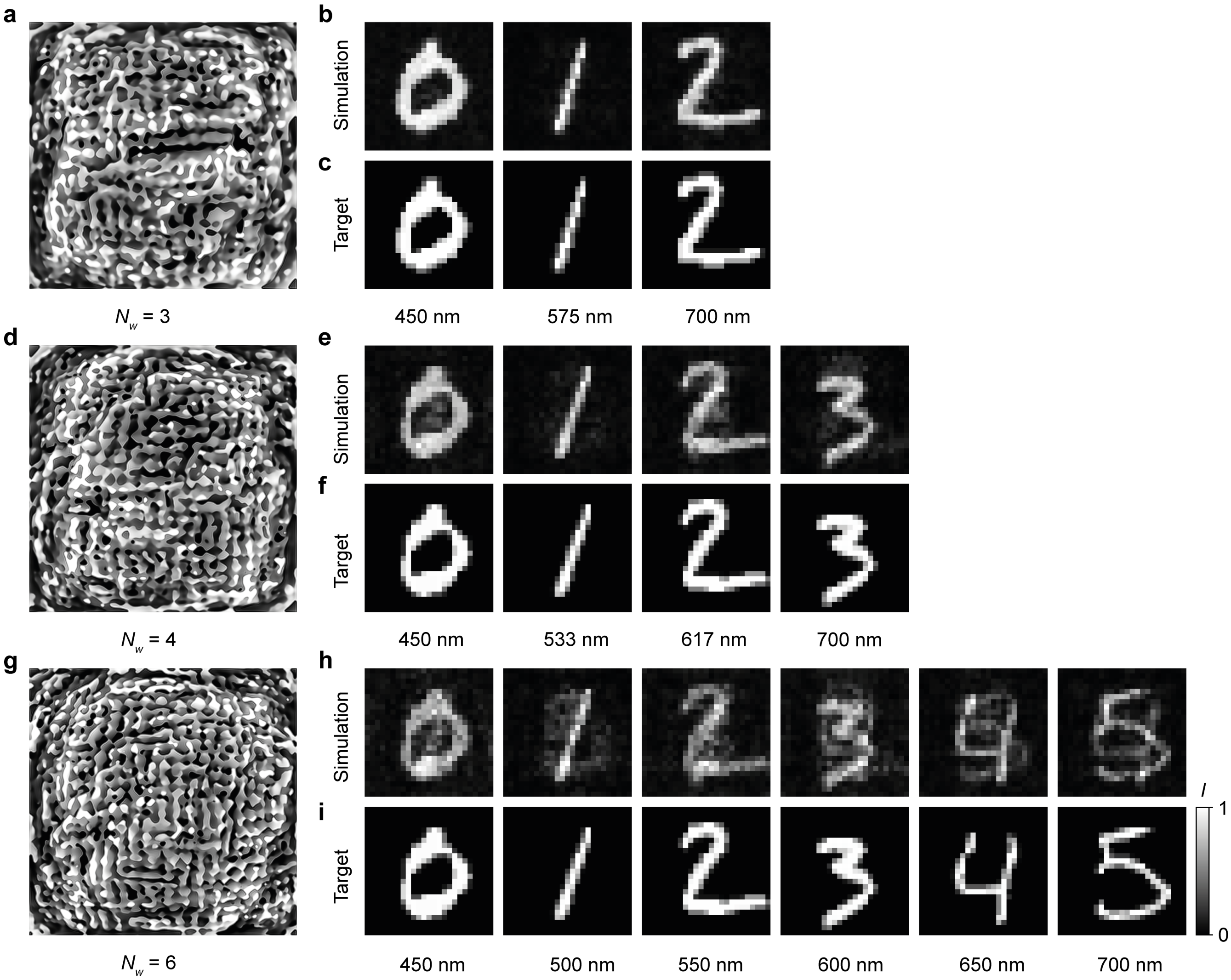

Figure S17. Impact of the number of wavelength channels N_w on the image reconstruction performance. (a),(d),(g) Grayscale hologram designs with different numbers of wavelength channels (N_w = 3, 4, and 6). The distance between the sample and image plane was fixed at d = 200 µm. Each hologram measures 50 µm × 50 µm with a pixel size of 40 nm × 40 nm (1250 × 1250 pixels, as in Figure S16 and Figure 4 in the main text). The encoded diffraction images are MNIST handwritten digits from 0 to $N_w - 1$. The wavelength channels are uniformly spaced between 450 and 700 nm. (b),(e),(h) Simulated intensity profiles compared with (c), (f), and (i) the corresponding target images. Intensities are normalized to the maximum value within each panel. As N_w increases, the wavelength channels become spectrally more closely spaced. While diffraction efficiency remains similar (due to the efficiency threshold in the loss function; see Section S1.8), image quality degrades due to enhanced crosstalk between the wavelength channels.

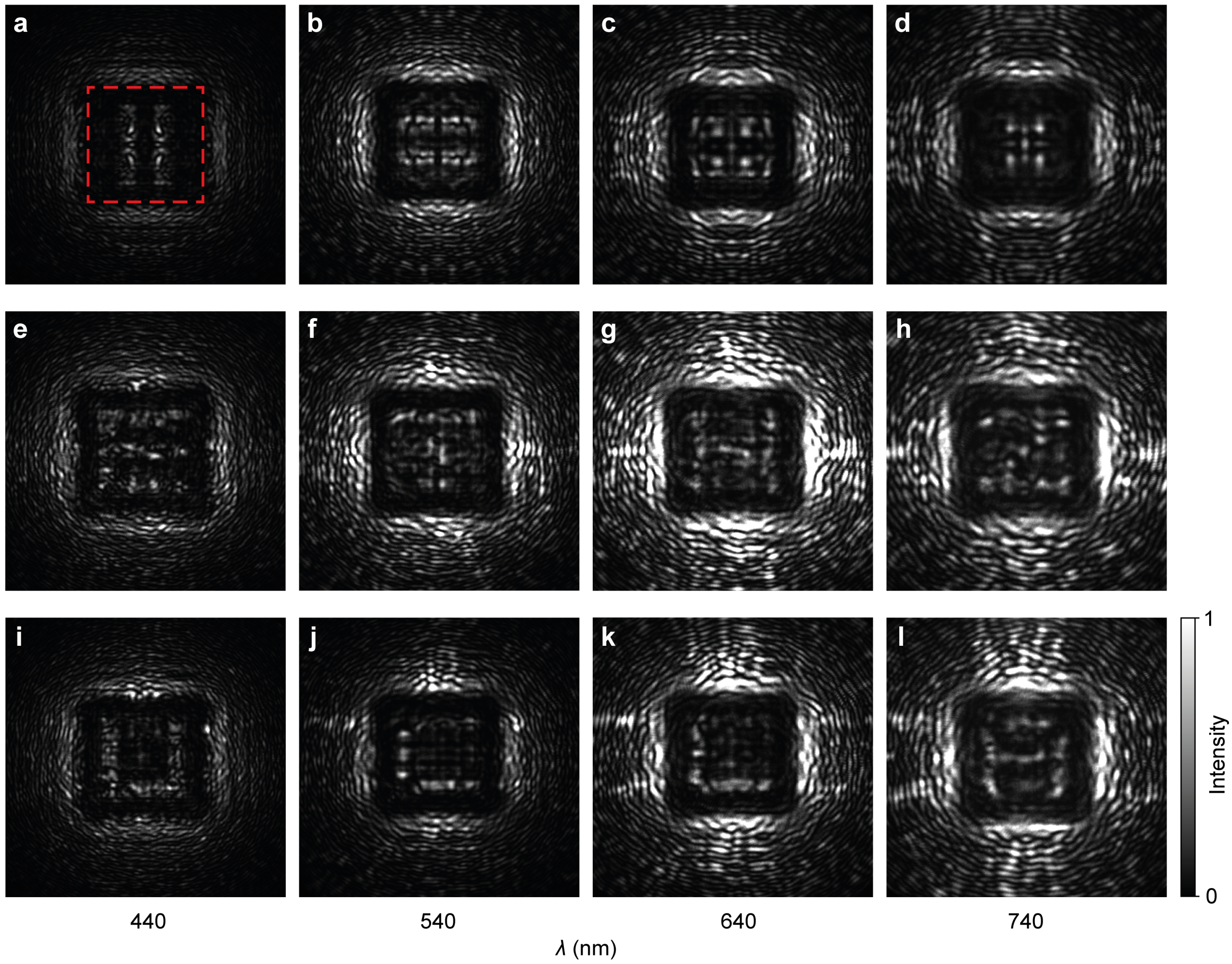

Figure S18. Raw experimental data for wavelength-multiplexed OFS holograms (N_w = 4). (a)–(l) Raw experimental intensity patterns for the holograms shown in Figure 5, designed to produce geometric shapes [squares and rectangles; (a)–(d)], the letters "ETHZ" (e)–(h), or the letters "UCLA" (i)–(l), respectively. Intensities are normalized to the maximum value within the field of view (FOV) of each panel (50 µm × 50 µm), indicated by the red dashed square in (a).

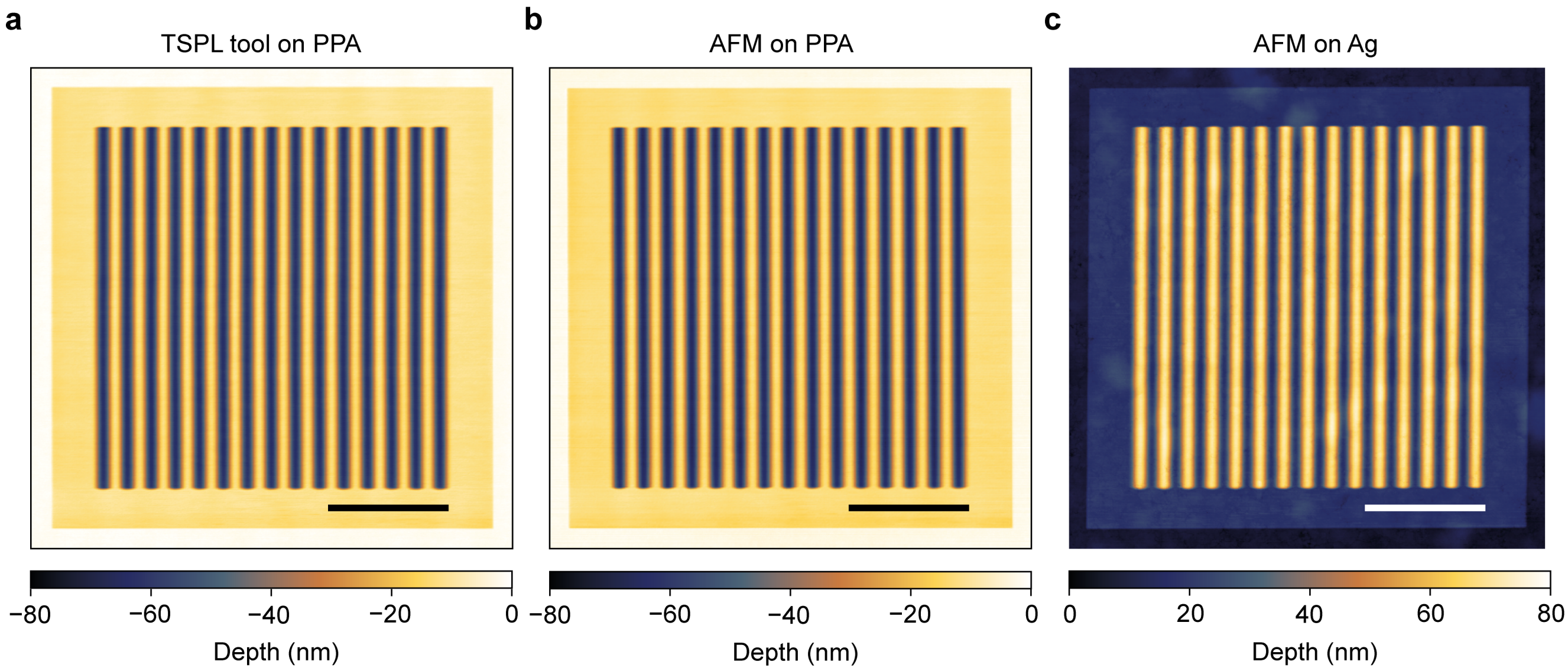

Figure S19. Topography characterization of a sinusoidal reference structure. (a) Topography data of the structure in PPA measured by the TSPL tool. (b) Surface profiles in PPA and (c) in Ag measured through atomic-force microscopy (AFM). The reference structure consists of a sinusoid (Λ = 600 nm, A = 25 nm) surrounded by a 1-μm-wide patterned frame. Scale bars represent 3 μm.

S3. SUPPORTING TABLES

Table S1. Efficiency and peak signal-to-noise (PSNR) of wavelength-multiplexed holograms (N_w = 3). Complementary data to Figure 4 in the main text.

MNIST digits	Simulation		Experiment	
Wavelength (nm)	Efficiency (%)	PSNR	Efficiency (%)	PSNR
450	8.6	20.4	8.4	10.8
575	4.3	22.2	8.7	17.5
700	6.3	20.4	10.4	13.1

Table S2. Efficiency and PSNR of wavelength-multiplexed holograms (N_w = 4). Complementary data to Figure 5 in the main text.

Geometric shapes	Simulation		Experiment	
Wavelength (nm)	Efficiency (%)	PSNR	Efficiency (%)	PSNR
440	11.8	20.1	10.4	10.2
540	7.4	20.2	12.7	11.0
640	6.8	19.5	9.2	9.9
740	7.6	19.9	7.8	10.2

ETHZ	Simulation		Experiment	
Wavelength (nm)	Efficiency (%)	PSNR	Efficiency (%)	PSNR
440	9.3	18.9	10.0	8.7
540	4.6	19.3	9.2	10.2
640	3.5	18.8	6.2	8.8
740	6.4	18.9	7.5	8.9

UCLA	Simulation		Experiment	
Wavelength (nm)	Efficiency (%)	PSNR	Efficiency (%)	PSNR
440	10.6	20.3	10.2	9.3
540	5.7	20.6	10.8	10.9
640	3.7	20.2	7.1	10.7
740	5.6	20.4	7.5	8.7

Table S3. Topography characterization of a sinusoidal reference structure. The statistical fitting uncertainties of the extracted periods Λ and amplitudes A are below 0.01 nm; these values describe the precision of the fit and should not be interpreted as the absolute metrological uncertainty of the AFM measurement.

	TSPL tool on PPA	AFM on PPA	AFM on Ag
Fit parameter	Value (nm)	Value (nm)	Value (nm)
Λ	601	605	604
A	24.9	26.0	27.3
RMSE	0.94	1.46	1.70

Table S4. Topography characterization of the different design strategies.

**Approach**	**Linear design**	**Gerchberg–Saxton**	**Gradient-based design**
**Intuition**	High	Low	Low
**Control**	Amplitude and phase	Amplitude	Flexible
**Efficiency**	Low	High	High
**Speckle / uniformity**	Uniformity directly specified by target field	Unconstrained phase results in speckles	Flexible
**Computation effort**	Low	Moderate	High
**Fabrication constraints**	Included through filtering/post-processing	Included through post-processing or algorithm constraints	Incorporated into optimization
**Multiplexing**	Straightforward through linear superposition	Possible through optimization	Flexible

REFERENCES

(S1) Glauser, Y. M.; Maris, J. J. E.; Brechbuhler, R.; Crimmann, J. G.; De Rosa, V. G.; Petter, D.; Nagamine, G.; Lassaline, N.; Norris, D. J. Diffraction of Light from Optical Fourier Surfaces. *ACS Photonics* **2025**, *12*, 2664–2675.

(S2) Glauser, Y. M.; Vonk, S. J. W.; Seda, D. B.; Niese, H.; de Jong, B.; Bidaut, M. F.; Bossavit, E.; Petter, D.; Nagamine, G.; Lassaline, N.; Norris, D. J. Fourier Pixels for Bidirectional Light Control. *Nature* **2026**, *655*, 87–92.

(S3) Goodman, J. W. *Introduction to Fourier Optics*; W. H. Freeman and Company, 2017.

(S4) Saleh, B. E. A.; Teich, M. C. *Fundamentals of Photonics*; John Wiley & Sons Inc, 2019.

(S5) Moreno, V.; Román, J. F.; Salgueiro, J. R. High Efficiency Diffractive Lenses: Deduction of Kinoform Profile. *Am. J. Phys.* **1997**, *65*, 556–562.

(S6) Harvey, J.; Pfisterer, R. Understanding Diffraction Grating Behavior: Including Conical Diffraction and Rayleigh Anomalies from Transmission Gratings. *Opt. Eng.* **2019**, *58*, 087105.

(S7) Harvey, J.; Pfisterer, R. Understanding Diffraction Grating Behavior, Part II: Parametric Diffraction Efficiency of Sinusoidal Reflection (Holographic) Gratings. *Opt. Eng.* **2020**, *59*, 017103.

(S8) Gerchberg, R. W. A Practical Algorithm for the Determination of Plane from Image and Diffraction Pictures. *Optik* **1972**, *35*, 237–246.

(S9) Pasienski, M.; DeMarco, B. A High-Accuracy Algorithm for Designing Arbitrary Holographic Atom Traps. *Opt. Express* **2008**, *16*, 2176–2190.

(S10) Fienup, J. Iterative Method Applied to Image Reconstruction and to Computer-Generated Holograms. *Opt. Eng.* **1980**, *19*, 297–305.

(S11) Fienup, J. R. Phase Retrieval Algorithms: A Comparison. *Appl. Opt.* **1982**, *21*, 2758–2769.

(S12) Li, J.; Gan, T.; Bai, B.; Luo, Y.; Jarrahi, M.; Ozcan, A. Massively Parallel Universal Linear Transformations Using a Wavelength-Multiplexed Diffractive Optical Network. *Adv. Photonics* **2023**, *5*, 016003.

(S13) Chang, C.; Xia, J.; Yang, L.; Lei, W.; Yang, Z.; Chen, J. Speckle-Suppressed Phase-Only Holographic Three-Dimensional Display Based on Double-Constraint Gerchberg–Saxton Algorithm. *Appl. Opt.* **2015**, *54*, 6994–7001.
